# A desert lncRNA *HIDEN* regulates human endoderm differentiation via interacting with IMP1 and stabilizing *FZD5* mRNA

**DOI:** 10.1186/s13059-023-02925-w

**Published:** 2023-04-24

**Authors:** Pei Lu, Jie Yang, Mao Li, Shanshan Wen, Tianzhe Zhang, Chenchao Yan, Ran Liu, Yu Xiao, Xinghuan Wang, Wei Jiang

**Affiliations:** 1 Department of Biological Repositories, Frontier Science Center for Immunology and Metabolism, Medical Research Institute, Zhongnan Hospital of Wuhan University, Wuhan University, Wuhan, 430071 China; 2 Department of Urology, Zhongnan Hospital of Wuhan University, Wuhan, 430071 China; 3 Human Genetics Resource Preservation Center of Wuhan University, Wuhan, 430071 China; 4 RNA Institute, Wuhan University, Wuhan, 430071 China

**Keywords:** Desert lncRNA, Human pluripotent stem cell, Endoderm differentiation, IMP1, FZD5

## Abstract

**Background:**

Extensive studies have revealed the function and mechanism of lncRNAs in development and differentiation, but the majority have focused on those lncRNAs adjacent to protein-coding genes. In contrast, lncRNAs located in gene deserts are rarely explored. Here, we utilize multiple differentiation systems to dissect the role of a desert lncRNA, *HIDEN* (**h**uman **I**MP1-associated "**d**esert" definitive **e**ndoderm l**n**cRNA), in definitive endoderm differentiation from human pluripotent stem cells.

**Results:**

We show that desert lncRNAs are highly expressed with cell-stage-specific patterns and conserved subcellular localization during stem cell differentiation. We then focus on the desert lncRNA *HIDEN* which is upregulated and plays a vital role during human endoderm differentiation. We find depletion of *HIDEN* by either shRNA or promoter deletion significantly impairs human endoderm differentiation. *HIDEN* functionally interacts with RNA-binding protein IMP1 (IGF2BP1), which is also required for endoderm differentiation. Loss of *HIDEN* or IMP1 results in reduced WNT activity, and WNT agonist rescues endoderm differentiation deficiency caused by the depletion of *HIDEN* or IMP1. Moreover, *HIDEN* depletion reduces the interaction between IMP1 protein and *FZD5* mRNA and causes the destabilization of *FZD5* mRNA, which is a WNT receptor and necessary for definitive endoderm differentiation.

**Conclusions:**

These data suggest that desert lncRNA *HIDEN* facilitates the interaction between IMP1 and *FZD5* mRNA, stabilizing *FZD5* mRNA which activates WNT signaling and promotes human definitive endoderm differentiation.

**Supplementary Information:**

The online version contains supplementary material available at 10.1186/s13059-023-02925-w.

## Background

Long noncoding RNAs (lncRNAs) are transcripts longer than 200 nucleotides without evident protein coding capacity. LncRNAs can cross-talk with DNA, RNA, and proteins, and exert biological function through chromatin remodeling, transcriptional or post-transcriptional gene regulation. The inspection of the genomic positions of lncRNA loci actively transcribed in human embryonic stem cells (ESCs) has revealed that 89% are associated with the promoters, enhancers, or bodies of protein-coding genes (PCGs) [1]. These lncRNAs could affect the expression of adjacent PCGs in a manner of base pair complementarity or acting as scaffolds to mediate local protein-DNA interaction [2]. However, the functional annotation of distal lncRNAs, especially those located in gene deserts (named as “desert lncRNAs”), is far less studied, mainly due to the difficulty of dissecting the biological function and revealing the downstream target.

Growing evidence has shown that lncRNAs are vital regulators in development and differentiation. LncRNAs participate in regulating lineage commitment and differentiation potential of pluripotent stem cells (PSCs), including neural differentiation, myogenesis, cardiogenesis, and endodermal lineage differentiation [3–5]. Definitive endoderm (DE), which arises at gastrulation when three germ layers emerge, is the innermost layer and later develops into the respiratory tract, the digestive tract, and their derivatives. During embryonic patterning and germ layer formation, high Activin or Nodal and modest WNT signaling together activate the DE program via the endoderm transcription factors *SOX17*, *FOXA2*, *EOMES* and *GATA4/6* [6–10]. Besides signaling pathways and transcription factors, epigenetic regulators and noncoding RNAs are also important contributors to precise and exquisite endoderm differentiation.

Most studies of endoderm lncRNAs focused on those physically located nearby lineage transcription factors, which illustrated the importance of the coordinated expression of lncRNA/mRNA gene pairs during development [1]. For instance, antisense lncRNA *Evx1as*, which can regulate EVX1 transcription through Mediator binding and chromatin looping, orchestrated mesendoderm differentiation of mouse ESCs [11]. *DEANR1* and *GATA6-AS1* facilitated SMAD2/3 binding on the promoter of its nearby PCG, *FOXA2* and *GATA6* respectively, to promote endoderm differentiation from human PSCs [12, 13]. Another lncRNA *DIGIT*, divergently transcribed from mesendoderm transcription factor GSC, regulated GSC expression to increase endoderm commitment [14]. A follow-up study further showed that *DIGIT* interacted with BRD3 and promoted phase-separated condensates of BRD3, which mediated BRD3 binding at H3K18ac-enriched promoter regions of endoderm transcription factors [15]. Of note, these reported endoderm-functional lncRNAs are all adjacent to PCGs and regulate the transcription of neighboring endoderm genes, thus contributing to endoderm differentiation. Nevertheless, whether and how desert lncRNAs located far away from PCGs regulate early germ layer differentiation is largely unknown. Besides, the biological roles of the evolution-extended desert lncRNAs in gene-poor regions need further detection but are often neglected due to the lack of large-scale screening methods and difficulty in mechanism study.

Our RNA-seq profiling of human ESCs and the derived purified DE cells revealed many DE-specific lncRNAs with unknown functions [12], including dozens of desert lncRNAs (i.e., without PCGs nearby in the genomic range of 50 kb). Here, we studied the function of a cytoplasm-localized desert lncRNA *HIDEN* (human IMP1-associated "desert" definitive endoderm lncRNA), which was highly expressed during human endoderm differentiation. Loss-of-function assay of *HIDEN* through shRNA or promoter deletion demonstrated that *HIDEN* was necessary for DE differentiation. We then identified the essential role of *HIDEN*-interacting protein IMP1 for DE differentiation. Further studies showed *HIDEN* physically interacted with IMP1 protein and together facilitated the mRNA stability of a WNT receptor Frizzled 5 (FZD5). Disruption of *HIDEN* resulted in lower *FZD5* mRNA expression and impaired WNT activation, which caused abnormal DE differentiation.

## Results

### Desert lncRNAs are highly expressed during human endoderm differentiation

**Fig. 1:**
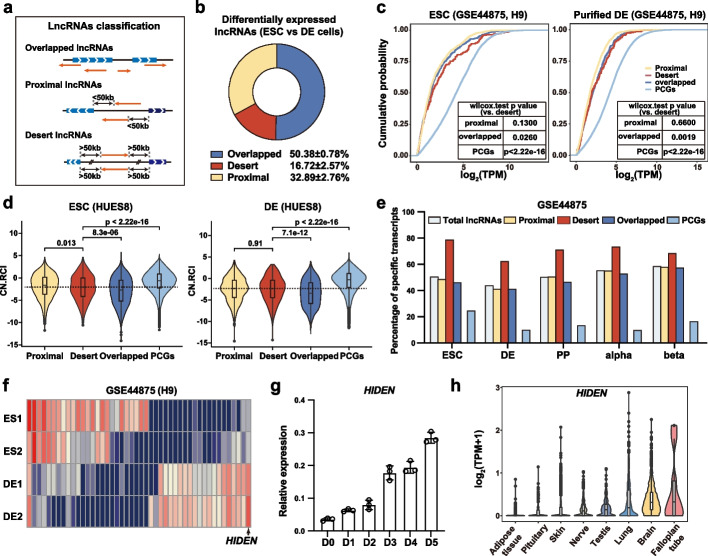
Desert lncRNAs are highly expressed during human endoderm differentiation. **a** The classification of overlapped lncRNAs, proximal lncRNAs and desert lncRNAs. **b** Pie chart showing the average proportion of differentially expressed overlapped, proximal, and desert lncRNAs identified between human PSCs and DE cells (data from three PSC lines). **c** The expression level of lncRNAs and PCGs in H9 ESCs and DE cells. The curves were colored by the category of lncRNAs. The *p* value between desert lncRNAs and other subsets were listed in the chart. **d** The subcellular localization of lncRNAs and PCGs, calculated by “relative concentration index” (RCI) in HUES8 ESCs and DE cells. CN.RCI = log_2_(CE/NE) + log_2_(CE/NM) (CE: cytoplasmic elution component, NE: nuclear elution component, NM: nuclear insoluble component). **e** The cell expression specificity of lncRNAs and PCGs in ESCs, DE, pancreatic endocrine (PP), pancreatic alpha and beta cells, calculated by specificity score. **f** Heatmap of differentially expressed desert lncRNAs between ESCs and DE cells. Red indicates higher expression while blue indicates lower expression. The lncRNAs list was shown in Additional file 2: Table S1. **g** Time course expression of *HIDEN* during endoderm differentiation from human HUES8 ESCs was detected by RT-qPCR (*n* = 3). **h** The expression of *HIDEN* in 30 human tissues from GTEx database. The top eight tissues with high expression were shown

To achieve a systematic understanding of the characteristics and functional relevance of lncRNAs in early embryo development, we first divided lncRNAs into three categories based on the genomic distance between lncRNAs and nearby PCGs from GENCODE V29 annotation [16]: the overlapped lncRNAs sharing at least one nucleotide with PCGs in the genome, the proximal lncRNAs locating close to PCGs within 50 kb but with no overlap with PCGs, and the desert lncRNAs being far away from PCGs more than 50 kb in the genome (Fig. 1a). We found that about half of lncRNAs were overlapped lncRNAs, while the percentage of proximal and desert lncRNAs was 27.92% and 23.72% separately (Additional file 1: Fig. S1a). All these lncRNAs showed minimal coding potential compared with PCGs (Additional file 1: Fig. S1b). To further explore the features of these lncRNAs, we systematically investigated the lncRNA expression level, subcellular localization and cell expression specificity by re-analyzing multiple RNA-seq data. We compared differentially expressed lncRNAs between ESCs and differentiated DE cells in three datasets (Additional file 2: Table S1), and about 16.72% were desert lncRNAs (Fig. 1b).

Compared to PCGs, all these lncRNAs showed lower RNA expression level in both PSCs and DE cells, but the desert lncRNAs were apparently higher than the other two (Fig. 1c, Additional file 1: Fig. S1c). Given that the function of lncRNAs is always linked to their subcellular localization [17, 18], we further dissected the subcellular localization of these lncRNAs based on the relative concentration index (RCI) [19]. The results showed that PCGs were mostly located in cytoplasm while lncRNAs showed significant nuclear localization tendency, especially for the overlapped lncRNAs (Fig. 1d, Additional file 1: Fig. S1d), which may be connected to its *cis*-regulatory model [1]. This nuclear localization tendency was also conserved in human lung cancer cell line A594 (Additional file 1: Fig. S1d). Previous reports showed that lncRNAs were expressed in a more cell-type-specific manner than PCGs [20]. Using a stage-specificity score [12], we found that lncRNAs exhibited more specific expression patterns not only in the continuous pancreatic lineage differentiation processes but also in different cell types, while desert lncRNAs showed the highest stage-specificity score among these lncRNAs (Fig. 1e). To further confirm the cell expression specificity of lncRNAs, we quantified the Normalized Difference (ND) of expression in the continuous pancreatic differentiation data [21]. The result was consistent with the stage-specificity score (Additional file 1: Fig. S1e). The cell-stage-specific expression feature of desert lncRNAs was also observed in another dataset of different cell types (Additional file 1: Fig. S1f-g). Altogether, these results indicated that lncRNAs were transcripts with low coding potential, nuclei-localized tendency, and significant cell-type-specific expression pattern.

Within all lncRNAs, the desert lncRNAs show higher expression level and significant cell-type-specific expression pattern, but the function and regulatory mechanism are largely uncharted in early embryo development. To explore the role of desert lncRNAs during human early differentiation, we identified the differentially expressed desert lncRNAs by transcriptome analysis of H9 ESC and its purified differentiated DE cells. Among the 46 differentially expressed desert lncRNAs (Additional file 2: Table S1), *HIDEN* (ENSG00000253507, AC104257.1) was highly expressed in DE cells (Fig. 1f). A time-course analysis demonstrated that *HIDEN* was gradually upregulated during DE differentiation (Fig. 1g), and with low coding potential predicted by two online software CPAT and CPC2 [22, 23] (Additional file 1: Fig. S1h). Furthermore, we analyzed the expression of *HIDEN* in 30 human tissues from the GTEx database and found *HIDEN* was highly expressed in germ lineages and brain as well as endoderm-derived lung (Fig. 1h).

### *HIDEN* is a desert lncRNA required for endoderm differentiation

To better annotate the transcript information of *HIDEN*, rapid amplification of cDNA ends (RACE) was performed to obtain the full-length and map the genomic location of *HIDEN* in HUES8-derived endoderm cells. Based on the 5’ and 3’ RACE results (Additional file 1: Fig. S2a), we cloned the full-length of *HIDEN* transcript from cDNA of HUES8 DE cells and *HIDEN* was annotated as 1045 nucleotides with four exons (sequences were shown in Additional file 3). Interestingly, there was another isoform in PGP1 cells (Additional file 1: Fig. S2b): 1169 nucleotides with one additional small exon (Additional file 3). The genome sequence of *HIDEN* exhibited low species conservation (Additional file 1: Fig. S2b).

**Fig. 2:**
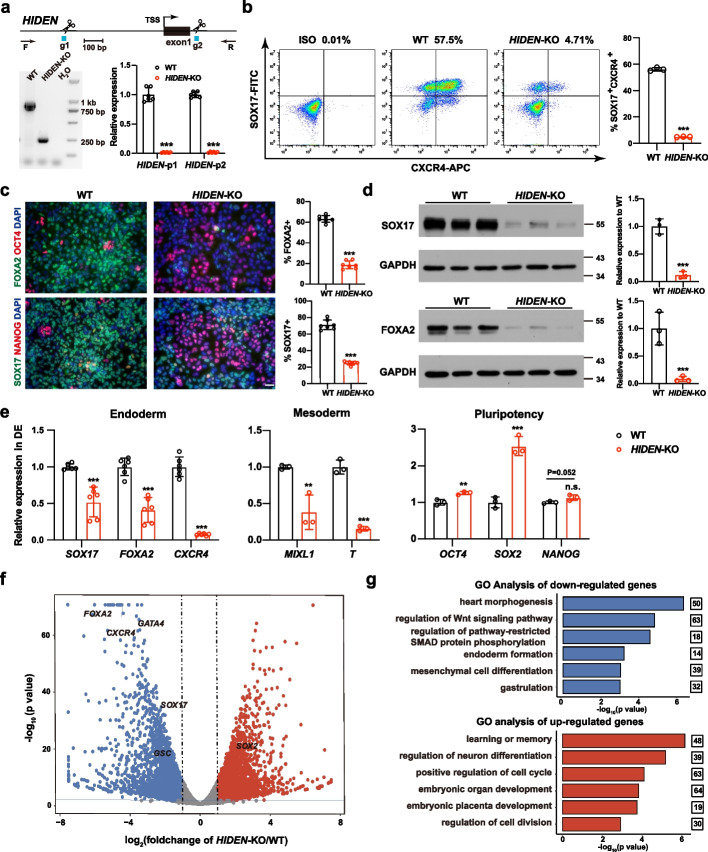
*HIDEN* is a desert lncRNA required for endoderm differentiation. **a** Generation of *HIDEN* knockout (KO) PSCs by CRISPR/Cas9. Top: sgRNAs used to delete the promoter of *HIDEN* and genomic PCR primers used to detect the promoter deletion. Bottom: The *HIDEN* RNA level was quantified using two sets of primers in *HIDEN*-KO DE cells compared to wildtype (*n* = 6). **b** Flow cytometric analysis of SOX17^+^CXCR4^+^ cells in wildtype and *HIDEN*-KO DE cells. The statistical results were shown on the right (*n* = 3). **c** Immunofluorescent staining of DE markers (SOX17, FOXA2) and pluripotency markers (OCT4, NANOG) in wildtype and *HIDEN*-KO DE cells. Quantitative results were shown on the right (*n* = 7). Scale bar, 50 $\upmu$m. **d** The protein levels of DE markers (SOX17 and FOXA2) were determined in wildtype and *HIDEN*-KO DE cells (*n* = 3). GAPDH was used as internal control. **e** The RNA expression levels of representative endoderm genes (*n* = 6), mesoderm genes (*n* = 3) and pluripotency genes (*n* = 3) in wildtype and *HIDEN*-KO DE cells. **f** Scatterplot showing differentially expressed genes identified by RNA-seq of wildtype and *HIDEN*-KO DE cells (*n* = 3). Upregulated and downregulated genes upon *HIDEN*-KO were shown in red and blue, respectively. **g** GO analysis of the downregulated or upregulated genes in *HIDEN*-KO DE cells compared to wildtype

To investigate the biological function of *HIDEN* during DE differentiation from human PSCs, we established a *HIDEN*-knockout cell line in PSC line PGP1 with dual-sgRNA guided CRISPR/Cas9 system by deleting the promoter and the first exon existing in both isoforms (Fig. 2a). PX459 plasmid was constructed to allow tandem expression of two sgRNAs whose target sites were indicated in Fig. 2a [24], and the co-expression of two sgRNAs enhanced chances of the fragment deletion between two sgRNA target sites. Genomic PCR and RT-qPCR results validated successful knockout and complete deletion of *HIDEN* (Fig. 2a). *HIDEN*-knockout PSCs maintained normal expression of pluripotency markers, such as OCT4 and SSEA4, as shown in immunofluorescent staining results (Additional file 1: Fig. S2c). RT-qPCR results further confirmed that the expression of pluripotency genes, such as *SOX2*, *OCT4* and *NANOG*, were not disrupted in *HIDEN*-knockout PSCs (Additional file 1: Fig. S2d). We also examined the cell proliferation based on cell counting kit (CCK) and the results showed *HIDEN*-knockout PSCs exhibited a comparable proliferation rate as wildtype PSCs (Additional file 1: Fig. S2e). Together, we demonstrated that the *HIDEN* deletion had no impact on pluripotent genes expression or self-renewal, indicating *HIDEN* was not required for pluripotency maintenance which was consistent with the low expression of *HIDEN* in undifferentiated state (Fig. 1g).

To determine whether *HIDEN* was essential for DE differentiation, we subjected the wildtype and *HIDEN*-knockout PSCs to DE differentiation. We then examined the endoderm differentiation efficiency by CXCR4 (CD184)- or SOX17-based flow cytometric analysis [25] and key endoderm gene expression (SOX17, FOXA2, CXCR4, GATA4/6) at day 3 [6–10]. In vitro endoderm differentiation was induced by activin signaling [25, 26], which usually produced cultures consisting of up to 60–80% definitive endoderm cells after 3 days’ differentiation in our system [13]. Flow cytometric analysis showed an obvious decrease of SOX17- and CXCR4- double positive endoderm cells in the *HIDEN*-knockout group: 4.71% in the knockout group compared to 57.5% in wildtype cells (Fig. 2b). Similarly, immunofluorescent staining results showed fewer FOXA2- and SOX17-positive and more OCT4- and NANOG-positive cells in *HIDEN*-deleted differentiated cells compared to wildtype (Fig. 2c). Western blot also indicated that the protein levels of SOX17 and FOXA2 were significantly decreased in *HIDEN* knockout differentiated cells (Fig. 2d). Furthermore, RT-qPCR assay showed the endoderm genes (*SOX17*, *FOXA2*, *CXCR4*) were much less activated and the RNA levels of pluripotency genes were not that rapidly downregulated in *HIDEN*-deleted differentiated cells compared with wildtype (Fig. 2e). Taken together, the deletion of *HIDEN* caused an obvious defect in endoderm differentiation of human PSCs.

We then investigated the transcriptome in *HIDEN*-deficient DE cells (Additional file 4: Table S2). Again, the transcript of *HIDEN* was completely gone based on RNA-seq data (Additional file 1: Fig. S2b). RNA-seq results identified 2844 downregulated genes and 2700 upregulated genes. The volcano plot displayed a remarkable decrease in expression of endoderm genes, such as *SOX17*, *FOXA2*, *CXCR4* and *GATA4* (Fig. 2f). In addition, gene ontology (GO) analysis of downregulated genes in *HIDEN*-knockout cells exhibited a significant enrichment in terms of heart morphogenesis, regulation of WNT signaling pathway, regulation of pathway-restricted SMAD protein phosphorylation, endoderm formation, mesenchymal cell differentiation and gastrulation (Fig. 2g, Additional file 5: Table S3). On the other hand, GO terms of upregulated genes in *HIDEN*-knockout cells included learning or memory, regulation of neuron differentiation, positive regulation of cell cycle, embryonic organ development, embryonic placenta development, and regulation of cell division (Fig. 2g, Additional file 5: Table S3), supporting that the deletion of *HIDEN* affected key signaling pathways and endoderm development process. These results further demonstrated that *HIDEN* was an important regulator of the endoderm-specific transcriptome.

To confirm the importance of *HIDEN* for endoderm differentiation, we conducted spontaneous embryonic bodies (EB) differentiation and RT-qPCR was used to quantify the expression of representative markers of three germ layers. Compared to wildtype cells, endoderm genes (*SOX17*, *FOXA2*, *CXCR4*) were decreased in *HIDEN*-knockout cells, while pluripotency genes (*OCT4*, *NANOG*), mesoderm genes (*MIXL1*, *T*) and ectoderm genes (*PAX6*, *SOX1*) were increased, which was consistent with the necessary role of *HIDEN* in DE differentiation (Additional file 1: Fig. S2f). In addition, we performed endodermal pancreatic differentiation in wildtype and *HIDEN-*deleted PGP1 cells with the previously reported protocol [27], including ESC, DE, pancreatic progenitor 1 (PP1), and pancreatic progenitor 2 (PP2) stages, to further confirm that *HIDEN* indeed was crucial for endoderm specification. The positive staining of key pancreatic marker PDX1 in both PP1 and PP2 were significantly reduced in *HIDEN-*deleted differentiated cells compared to wildtype cells (Additional file 1: Fig. S2g). Consistently, the RNA expression of pancreas-specific transcription factors was also decreased in *HIDEN-*deleted differentiated cells, such as *PDX1*, *FOXA2*, *GATA6* in PP1 stage and *PDX1*, *NKX6-1*, *PTF1A*, *NKX2-2*, *NGN3* in PP2 stage (Additional file 1: Fig. S2h). These results illustrated the pivotal role of *HIDEN* in human definitive endoderm differentiation*.*

To further confirm the necessary role of *HIDEN* in DE differentiation, we generated two stable cell lines with *HIDEN* knockdown in HUES8 using short hairpin RNAs (shRNAs), achieving at least 63% efficiency (Additional file 1: Fig. S3a). Pluripotent markers, such as SOX2, OCT4, NANOG and SSEA4, showed no significant difference between control and *HIDEN*-KD ESCs demonstrated by either RT-qPCR or immunofluorescence results (Additional file 1: Fig. S3b-c). Consistently, we observed that *HIDEN* knockdown severely impaired human ESC differentiation toward DE (Additional file 1: Fig. S3d-g).

### *HIDEN* physically interacts with IMP1

**Fig. 3:**
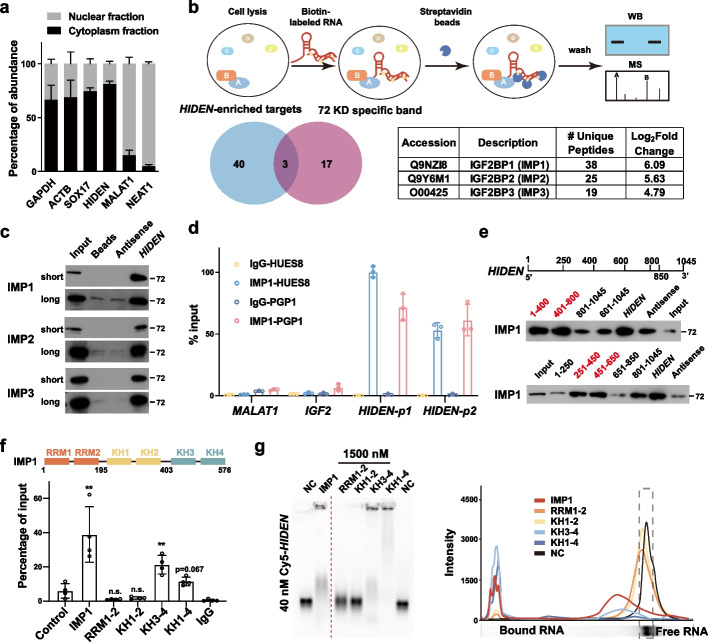
*HIDEN* physically interacted with IMP1. **a** Subcellular localization of *HIDEN* in DE cells determined by RT-qPCR following nucleo-cytoplasmic separation (*n* = 6). **b** The schematic diagram of RNA pulldown was shown on the top. Venn diagram indicates the overlapped hints identified by mass spectrometry of the specific band around 72 kDa (unique peptides ≥ 4, red circle) and whole extracts (unique peptides ≥ 10 in *HIDEN-*pulldown group and log_2_(fold-change (*HIDEN*/Antisense)) > 2.32, blue circle). The unique peptides and log_2_(fold-change) of IMP1/2/3 compared to antisense in mass spectrometry data of *HIDEN*-pulldown group were shown in the table. **c** Immunoblot for IMP1, IMP2, and IMP3 after RNA pulldown in DE cells. Beads and antisense were used as negative controls. Pictures captured for short and long exposure time were shown. **d** IMP1 RIP followed by RT-qPCR in DE cells of two PSC lines, PGP1 and HUES8 (*n* = 3). RNA levels were normalized to input. **e** Mapping the IMP1-binding region in *HIDEN* in 293 T cells. Top, diagrams of full-length *HIDEN* and the deletion fragments used in RNA pulldown. Bottom, immunoblot for IMP1 in protein samples pulled down by different *HIDEN* fragments. **f** Mapping the *HIDEN*-binding domain in IMP1 protein. Domain structure of IMP1 protein (top). FLAG-tagged IMP1 or IMP1 mutants and *HIDEN* were co-overexpressed in 293 T cells and FLAG RIP was performed to examine the enrichment of *HIDEN* (bottom). **g** Electrophoretic mobility shift assay (EMSA) results of in vitro binding assays. Cy5-labeled *HIDEN* RNA (40 nM) were transcribed in vitro and His-tagged IMP1 proteins (1500 nM) were purified from *E. coli*

Next, we questioned how *HIDEN* contributed to endoderm differentiation from human PSCs, starting with determination of *HIDEN* expression in different cellular fractions in DE cells. While nuclear-localized *MALAT1* and *NEAT1* and cytoplasm-localized *GAPDH*, *ACTB*, and *SOX17* mRNAs showed expected subcellular localization in our assay, we found *HIDEN* was mainly localized in the cytoplasmic fraction of differentiated endoderm cells (Fig. 3a), implying that *HIDEN* probably regulated gene expression at the post-transcriptional level by interacting with RNA-binding proteins.

To identify the interacting proteins of *HIDEN*, we conducted RNA pull-down assay using biotin-labeled *HIDEN* or its antisense RNA or other unrelated RNAs including *luciferase* and *GATA6-AS1* as controls in DE cells (Fig. 3b). Silver staining showed an enriched band around 72 kDa captured specifically by *HIDEN* (Additional file 1: Fig. S4a), so this specific band was cut for mass spectrometry analysis (Additional file 6: Table S4). Meanwhile, whole immunoprecipitated extracts of *HIDEN* and its antisense were sent for quantitative mass spectrometry analysis and fold-change of *HIDEN*/antisense was calculated (Additional file 6: Table S4). The overlapped hints of mass spectrometry data based on the specific 72 kDa band and whole IP extracts were IGF2BP1 (IMP1), IGF2BP3 (IMP3), and IGF2BP2 (IMP2) (Fig. 3b). Western blot following RNA pulldown also confirmed the interaction of *HIDEN* with IMP1, IMP2, and IMP3 in DE cells (Fig. 3c). All these three proteins belong to the IMP family and are highly conserved RNA-binding proteins. Given that IMP1 was the most enriched protein in mass spectrometry analysis (Fig. 3b), we decided to take IMP1 for further study. RIP-qPCR analysis showed IMP1 was significantly bound with *HIDEN*, rather than mRNA *IGF2* or lncRNA *MALAT1* in DE cells from both PSC lines (Fig. 3d). These results indicated that *HIDEN* physically interacted with IMP1 protein.

To explore the direct binding region of *HIDEN* for IMP1, we used RNAfold (http://rna.tbi.univie.ac.at/cgi-bin/RNAWebSuite/RNAfold.cgi) and Mfold (http://www.unafold.org) to model the secondary structure of *HIDEN*. We obtained a similar secondary structure model of *HIDEN* from both tools (Additional file 1: Fig. S4b), and based on the predicted structure, we roughly divided *HIDEN* into three fragments: 1–400 nt, 401–800 nt and 801–1045 nt. We accordingly generated different biotin-labeled truncated *HIDEN* fragments for RNA pulldown assay in HEK293T cells. The result indicated that 1-400 nt and 401-800 nt fragments of *HIDEN* were responsible for the interaction between *HIDEN* and IMP1 (Fig. 3e). The second round of deletion-mapping analysis using smaller fragments of *HIDEN* revealed that two regions (251-450nt, 451-650nt) were mainly responsible for the interaction of *HIDEN* with IMP1 (Fig. 3e). IMP1 protein is composed of six canonical RNA-binding domains, including two RNA recognition motif (RRM) domains and four K homology (KH) domains (Fig. 3f). To clarify which domain of IMP1 interacted with *HIDEN*, we performed the co-expression experiment of truncated FLAG-tagged IMP1 and *HIDEN* in HEK293T cells. The RIP-qPCR results showed the highest enrichment of *HIDEN* in truncated KH3-4 domain compared to other mutants (Fig. 3f). We also purified various truncated IMP1 proteins (Additional file 1: Fig. S4c) and incubated with full-length Cy5-labeled *HIDEN* transcript for electrophoretic mobility shift assay (EMSA). Consistently, EMSA results showed that the KH3-4 domains of IMP1 were necessary for IMP1 binding to *HIDEN* at different concentrations (Fig. 3g and Additional file 1: Fig. S4d). Taken together, the 251–650 nt fragment of *HIDEN* and the KH3-4 domains of IMP1 were identified to be responsible for the interaction between *HIDEN* and IMP1 protein separately.

### IMP1 deficiency inhibits endoderm differentiation

As the role of the *HIDEN*-interacting protein IMP1 in endoderm differentiation was unknown, we next examined whether IMP1 functioned in endoderm differentiation as well. We performed a loss-of-function assay by CRISPR/Cas9 in H9 ESCs and finally generated three IMP1 knockout clones with different genotypes (Additional file 1: Fig. S5a), which displayed normal colony morphology (Additional file 1: Fig. S5b). We confirmed the complete loss of IMP1 protein in these IMP1-knockout cell lines by immunoblot (Additional file 1: Fig. S5c). The expression of pluripotency genes (*SOX2*, *NANOG*, and *OCT4*) was almost unchanged in IMP1-knockout ESCs compared to wildtype (Additional file 1: Fig. S5d).

**Fig. 4:**
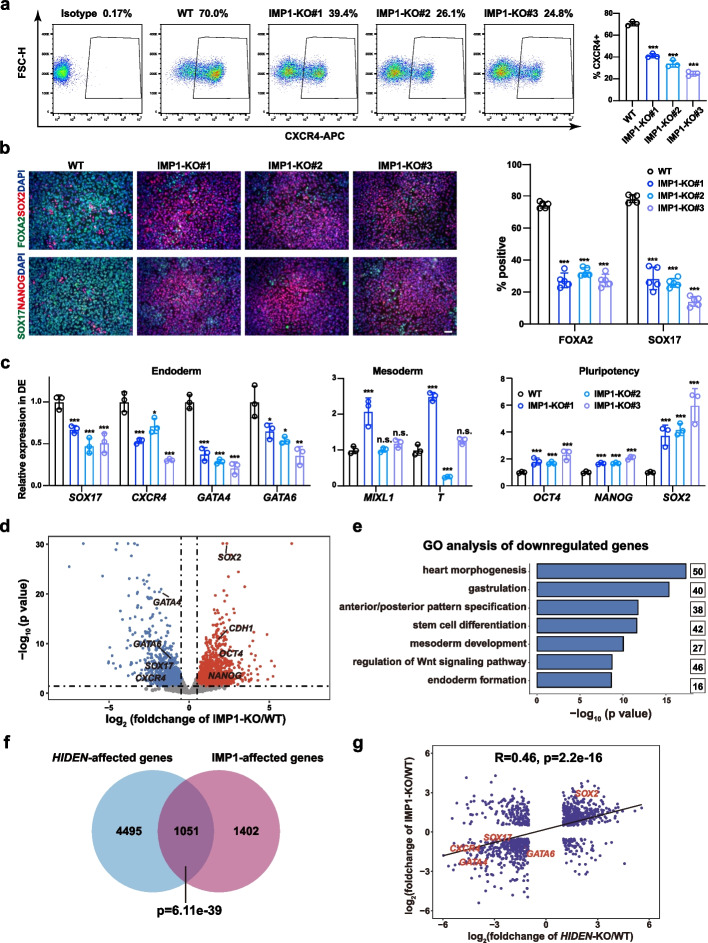
IMP1 deficiency inhibits endoderm differentiation. **a** Flow cytometric analysis of CXCR4-positive cells in wildtype (WT) and IMP1-KO DE cells. The statistical results were shown on the right (*n* = 3). **b** Immunofluorescent staining of DE markers (SOX17, FOXA2) and pluripotency markers (NANOG, SOX2) in WT and IMP1-KO DE cells. Quantitative results were shown on the right (*n* = 5). Scale bar = 50 μm. **c** RNA levels of representative endoderm genes, mesoderm genes and pluripotency genes in WT and IMP1-KO DE cells determined by RT-qPCR (*n* = 3). **d** Scatterplot showing differentially expressed genes in RNA-seq of WT and IMP1-KO DE cells. Upregulated and downregulated genes upon IMP1 knockout were shown in red and blue, respectively. **e** GO analysis of the downregulated genes in DE cells upon IMP1 knockout. **f** The overlap of differentially expressed genes upon *HIDEN*-KO and IMP1-KO. **g** The correlation of *HIDEN*-KO and IMP1-KO affected genes

Then, we subjected these IMP1-knockout ESCs to endoderm differentiation. Flow cytometric results showed a significant decrease of CXCR4-positive cells in IMP1-knockout cells (Fig. 4a). Consistently, immunostaining results revealed that IMP1 knockout resulted in reduced expression of endoderm markers (FOXA2, SOX17) and remaining expression of pluripotency markers (SOX2, NANOG) (Fig. 4b). Moreover, RT-qPCR results showed a consistent decreased expression of endoderm genes, such as *SOX17*, *CXCR4*, *GATA4* and *GATA6*, along with higher pluripotency genes expression (*OCT4*, *NANOG*, *SOX2*) and disrupted mesoderm genes expression (*MIXL1*, *T*), in IMP1-knockout DE cells (Fig. 4c). In addition, we performed RNA-seq analysis of wildtype and IMP1-knockout DE cells (Additional file 4: Table S2). From the volcano plot, we observed the downregulated endoderm genes (*SOX17*, *CXCR4*, *GATA4*, *GATA6*) and upregulated pluripotency genes (*SOX2*, *OCT4*, *NANOG*) (Fig. 4d). The top GO terms of downregulated genes in IMP1-deficient differentiated cells were heart morphogenesis, gastrulation, anterior/posterior pattern specification, stem cell differentiation, regulation of WNT signaling pathway, endoderm formation (Fig. 4e, Additional file 5: Table S3), similar to the results in *HIDEN*-knockout endoderm cells (Fig. 2g). We next compared the differentially expressed genes upon *HIDEN*- or IMP1-knockout identified by transcriptome analysis. We found 1051 genes co-regulated by *HIDEN*/IMP1, including endoderm genes such as *SOX17*, *GATA6*, *GATA4* and *CXCR4* (Fig. 4f). Moreover, the linear correlation result of co-regulated genes by *HIDEN*/IMP1 indicated *HIDEN* and IMP1 acted in the same gene regulatory loop during DE differentiation (Fig. 4g).

To further exclude the off-target effect of CRISPR/Cas9 and confirm the important role of IMP1 in endoderm differentiation, we generated three stable IMP1-knockdown ESC lines using shRNAs (Additional file 1: Fig. S5e). These IMP1-knockdown ESCs exhibited obvious endoderm differentiation defects in RNA expression of endoderm markers (*SOX17*, *FOXA2*, *CXCR4*) (Additional file 1: Fig. S5f) and the percentage of CXCR4 positive cells (Additional file 1: Fig. S5g). Overall, these results indicated that the depletion of IMP1 resulted in impaired endoderm differentiation from human PSCs.

### WNT signaling pathway acts as downstream of *HIDEN*/IMP1

**Fig. 5:**
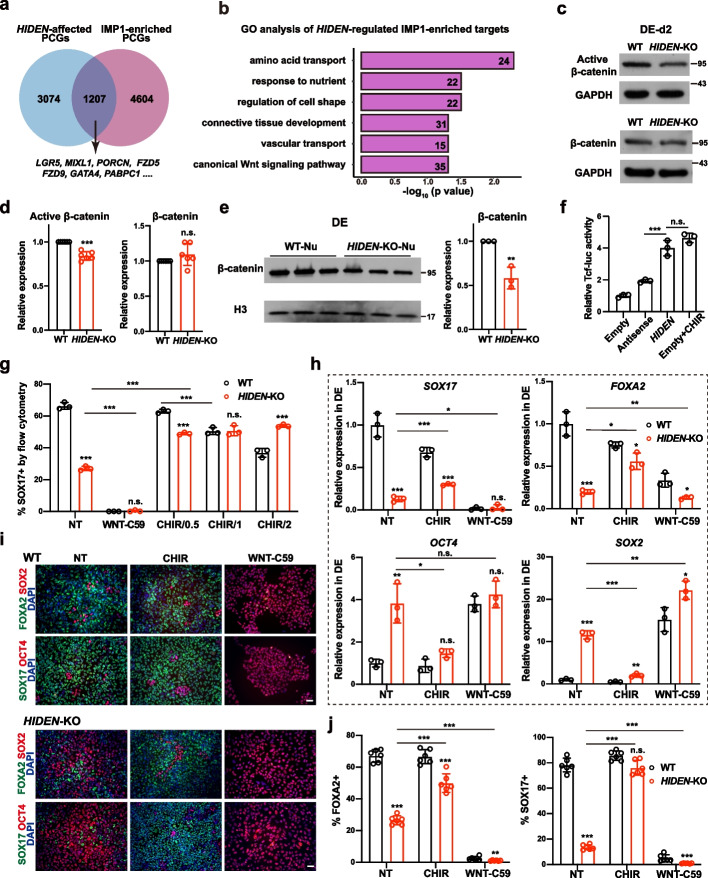
WNT signaling pathway acts as downstream of *HIDEN*/IMP1. **a** Venn diagram indicates the overlapped genes of differentially expressed PCGs upon *HIDEN* knockout in DE cells and IMP1-enriched targets identified by IMP1 RIP-seq in DE cells. **b** GO analysis of the overlapped genes from **a**. **c**,** d ***HIDEN* knockout led to reduced active β-catenin and unaltered total β-catenin level compared to wildtype after two days’ DE differentiation, as shown by Western blot (**c**) and statistical results (**d**) (*n* = 6). **e** The protein level of β-catenin in nuclear fraction of WT or *HIDEN*-KO DE cells (*n* = 3). **f** The TCF-luciferase activity in 293 T when transfected with *HIDEN*, antisense control or empty vector (*n* = 3). Cells treated with 1 μM CHIR-99021 were used as positive controls. **g-j** Flow cytometric analysis of SOX17-positive cells (**g**), the presentative endoderm genes expression revealed by RT-qPCR (**h**) and immunostaining (**i-j**) in wildtype or *HIDEN*-KO cells after manipulating WNT signaling through small molecular inhibitors during DE differentiation (*n* = 3). WNT signaling activator CHIR-99021, with different concentrations (0.5 μM, 1 μM and 2 μM) at (**g**) and 1 μM at (**h-j**), and WNT signaling inhibitor WNT-C59 (1 μM) were used. NT indicated for non-treated group. **i** Scale bar = 50 μm. **j** Quantitative results of SOX17- and FOXA2-positive cells were shown (*n* = 6)

To understand how *HIDEN* and IMP1 affected human endoderm differentiation, we first examined the expression of IMP1 upon *HIDEN* depletion. The protein level of IMP1 remained unchanged in *HIDEN*-knockout or -knockdown DE cells compared to control cells (Additional file 1: Fig. S6a-b). Since IMP1 is a conserved RNA-binding protein and regulates mRNA stability, translation efficiency or cellular localization [28, 29], we examined the RNA-binding capacity of IMP1 in *HIDEN* knockout DE cells by RIP-seq. We integrated RNA-seq and RIP-seq data and identified the candidate target genes of *HIDEN*/IMP1 by overlapping the IMP1-bound RNAs and the differentially expressed protein-coding genes in DE cells upon *HIDEN* deletion (Fig. 5a). GO analysis of *HIDEN*/IMP1 target genes enriched in terms of nutrient response and transport, regulation of cell shape, connective tissue development, and canonical WNT signaling pathway (Fig. 5b, Additional file 5: Table S3). More importantly, by comparing IMP1-bound between wildtype and *HIDEN*-knockout DE cells, we identified 4880 genes with reduced IMP1 binding upon depleting *HIDEN* (Additional file 1: Fig. S6c, Additional file 7: Table S5). The GO analysis of these genes enriched terms of regulation of mRNA metabolic process, RNA splicing, WNT signaling pathway, canonical WNT signaling pathway, stem cell population maintenance, and endoderm development (Additional file 1: Fig. S6d, Additional file 5: Table S3). Considering the importance of the WNT signaling pathway in endoderm differentiation [7, 30, 31] and the fact that the enriched GO term of regulation of WNT signaling pathway upon *HIDEN* or IMP1 knockout, we hypothesized that WNT pathway acted as the downstream of *HIDEN*/IMP1. Therefore, we collected wildtype or *HIDEN*-deleted DE cells during endoderm differentiation to evaluate the protein level of active (unphosphorylated, nuclear-located) β-catenin, the key effector of WNT signal pathway. Compared to wildtype cells, we observed a significant decrease of active β-catenin and unaltered expression of total β-catenin at both differentiation day 2 (Fig. 5c, d) and day 4 (Additional file 1: Fig. S6e) in *HIDEN*-knockout cells. Consistently, β-catenin was significantly reduced in the nuclear fraction (Fig. 5e), which further proved the reduced WNT signaling activity in *HIDEN*-depleted DE cells. We also observed the similarly decreased active β-catenin in differentiated *HIDEN*-knockdown cells (Additional file 1: Fig. S6f). In addition, we performed the β-catenin/TCF-responsive luciferase reporter assay in HEK293T cells, showing that the overexpression of *HIDEN* led to elevated TCF luciferase activity (Fig. 5f). These results suggested that *HIDEN* depletion indeed resulted in impaired WNT activity during endoderm differentiation of PSCs.

We next explored whether the reduced WNT signaling activity accounted for the decreased endoderm differentiation caused by *HIDEN* deletion. WNT signal activation is necessary for highly efficient DE differentiation, especially for the early phase of mesendoderm differentiation [30, 32, 33]. Therefore, we applied WNT activator CHIR-99021 and inhibitor Wnt-C59 respectively to rescue or phenocopy the defected DE differentiation upon *HIDEN* deletion. Indeed, we observed CHIR treatment could elevate the SOX17-positive cells specifically in *HIDEN*-knockout cells (Fig. 5g), while Wnt-C59 treatment significantly impeded endoderm differentiation in both groups (Fig. 5g). RT-qPCR results also showed increased endoderm gene expression (*SOX17*, *FOXA2*) in CHIR-treated *HIDEN*-deleted DE cells compared to the non-treated group (Fig. 5h), along with decreased pluripotency gene expression (*OCT4*, *SOX2*) (Fig. 5h) and disrupted mesoderm gene expression (*MIXL1*, *T*) (Additional file 1: Fig. S6g). Wnt-C59 treatment led to severe blocked DE differentiation in both wildtype and *HIDEN*-deleted DE cells, indicated by low expression of endoderm genes, high expression of pluripotency genes (Fig. 5h). Immunostaining results showed similar results (Fig. 5i-j). These results together indicated that manipulating WNT signaling pathway could partially rescue the endoderm differentiation deficiency caused by *HIDEN* disruption, indicating WNT signaling pathway acted as the functional downstream of *HIDEN* to regulate endoderm differentiation.

We also tested the functional link between IMP1 and WNT signaling pathway in endoderm differentiation. Similar to *HIDEN*-knockout cells, the WNT activator CHIR or inhibitor Wnt-C59 was able to rescue or phenocopy the failed DE differentiation due to IMP1 knockout, respectively. Flow cytometry analysis exhibited increased CXCR4-positive DE cells in CHIR-treated IMP1-deleted DE cells compared to the non-treated group (Additional file 1: Fig. S6h). Consistent with this functional assay, overexpression of IMP1 could activate the transcriptional activity of β-catenin/TCF-responsive luciferase reporter (Additional file 1: Fig. S6i). Taken together, these results supported that WNT signaling pathway largely contributed to *HIDEN*/IMP1-mediated endoderm regulation.

### *HIDEN*/IMP1 promotes WNT signal pathway through FZD5

**Fig. 6:**
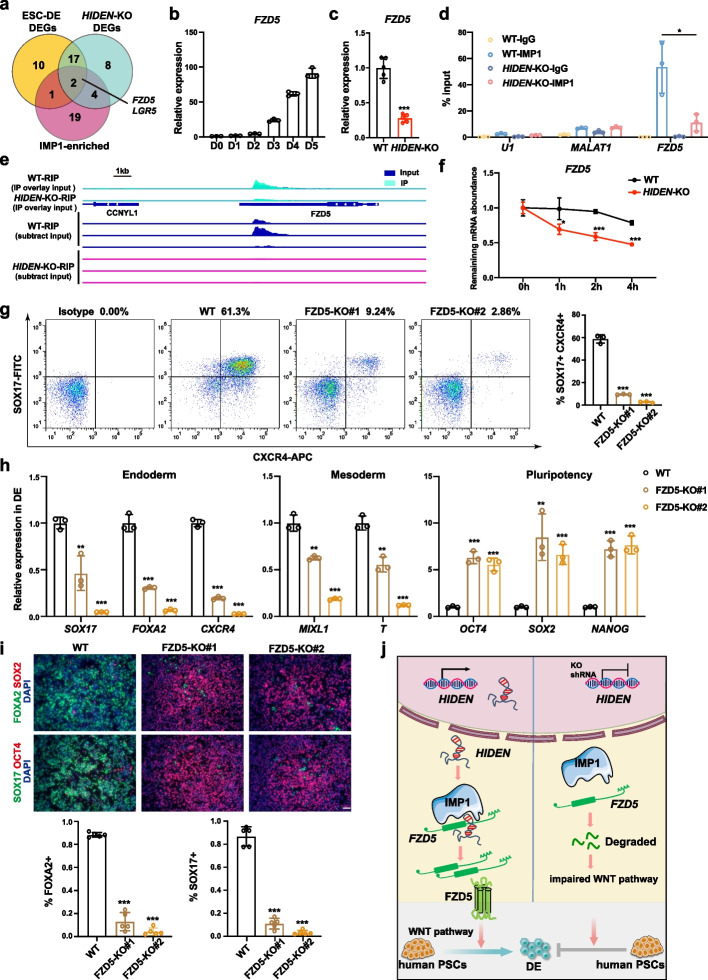
*HIDEN*-IMP1 stabilized *FZD5* mRNA to contribute to DE differentiation. **a** Venn diagram indicated the overlapping of WNT-associated differentially expressed genes during DE differentiation (yellow circle), WNT-associated *HIDEN*-regulated genes (green circle), and IMP1-enriched WNT-associated genes (pink circle). The WNT-associated genes were listed in Additional file 8: Table S6 (from online WNT website: http://web.stanford.edu/group/nusselab/cgi-bin/wnt/). **b** The time course expression of *FZD5* during DE differentiation, as shown by RT-qPCR (*n* = 3). **c** The relative expression of *FZD5* in wildtype or *HIDEN*-KO DE cells was determined by RT-qPCR (*n* = 3). **d** RT-qPCR following IMP1 RIP was performed for determination of the indicated transcripts enrichment by IMP1 in wildtype or *HIDEN*-KO DE cells (*n* = 3). RNA levels were normalized to input. *U1* and *MALAT1* were considered as negative controls. **e** The visualization of IMP1 binding on *FZD5* mRNA identified by IMP1 RIP-seq in wildtype or *HIDEN*-knockout DE cells. A detailed description was in Methods. **f** RNA stability assay of *FZD5* in wildtype or *HIDEN*-KO DE cells treated with Actinomycin D (*n* = 3). **g** Flow cytometric analysis of SOX17^+^CXCR4^+^ cells in wildtype or FZD5-KO DE cells (*n* = 3). **h** RNA levels of representative endoderm genes, mesoderm genes and pluripotency genes in wildtype or FZD5-KO DE cells, determined by RT-qPCR (*n* = 3). **i** Immunofluorescent staining of DE markers (SOX17, FOXA2) and pluripotency markers (OCT4, SOX2) in wildtype or FZD5-KO DE cells. Quantitative results of SOX17- and FOXA2-positive cells were shown on the bottom (*n* = 5). Scale bar = 50 μm. **j** The functional model of *HIDEN* in human DE differentiation

To find out the specific *HIDEN*/IMP1 direct targets to exert WNT-promoting effects, we studied the WNT-associated genes (listed in Additional file 8: Table S6), by the overlap among differentially expressed genes during DE differentiation (Additional file 1: Fig. S7a), differentially expressed genes upon *HIDEN* deletion in DE cells (Additional file 1: Fig. S7b), and IMP1-bound genes defined by IMP1 RIP-seq in DE cells (Fig. 6a). The overlapped genes were *FZD5* and *LGR5* (Fig. 6a). The RNA-seq analysis indicated *FZD5* and *LGR5* exhibited increased expression during endoderm differentiation and were downregulated upon *HIDEN* knockout (Additional file 1: Fig. S7c-d). Since the expression level of *LGR5* was relatively lower than *FZD5* during DE differentiation (Additional file 1: Fig. S7c-d), we mainly focused on *FZD5*. The upregulation of *FZD5* expression during DE differentiation from human PSCs and the downregulation of *FZD5* expression upon *HIDEN* deletion in endoderm cells were confirmed by RT-qPCR results (Fig. 6b, c). More importantly, IMP1 could highly enrich *FZD5* mRNA in DE cells, and this interaction between IMP1 protein and *FZD5* mRNA largely depended on *HIDEN* (Fig. 6d). Compared to differentiated wildtype cells, the binding of IMP1 to *FZD5* mRNA was significantly decreased and the expression of *FZD5* was much lower upon *HIDEN* knockout (Fig. 6e). The relative higher enrichment of *FZD5* mRNA pulled down by *HDIEN* compared to other controls, such as lncRNA *MALAT1*, mRNA *GAPDH* and *IMP1* (Additional file 1: Fig. S7e), further proved the association of *HIDEN* and *FZD5* mRNA. By transcription inhibition assay using Actinomycin D treatment, we found *FZD5* mRNA was more destabilized in *HIDEN*-knockout DE cells compared to wildtype cells (Fig. 6f). In addition, upon IMP1 knockout, we observed the lower expression of *FZD5* mRNA in differentiated cells (Additional file 1: Fig. S7f), indicating the role of *HIDEN* in regulating the stability of *FZD5* mRNAs via IMP1.

To determine whether the *HIDEN*/IMP1-regulated FZD5 was the functional target in DE differentiation, we generated two FZD5 knockout human ESC lines in HUES8 (Additional file 1: Fig. S7g) which showed remarkably decreased expression of *FZD5* (Additional file 1: Fig. S7h). Importantly, when subjected to endoderm differentiation conditions, FZD5 knockout led to blocked endoderm differentiation, evidenced by the reduced endoderm markers in flow cytometry analysis (Fig. 6g), RT-qPCR (Fig. 6h), and immunostaining results (Fig. 6i). In addition, the knockdown ESCs by shRNAs targeting *FZD5*, albeit maintained the normal expression of pluripotency genes demonstrated by immunofluorescence results (Additional file 1: Fig. S7i-j), indeed resulted in impaired endoderm differentiation, which was indicated by reduced endoderm gene expression in immunostaining, flow cytometry analysis, and RT-qPCR results (Additional file 1: Fig. S7k-m). These results suggested that the decreased FZD5 expression caused by *HIDEN*/IMP1 depletion inhibited endoderm differentiation from human PSCs.

## Discussion

In this study we have characterized a subclass of lncRNAs (“desert” lncRNAs) and investigated the biological role and underlying mechanism of a specific desert lncRNA *HIDEN* in human endoderm differentiation (Fig. 6j). *HIDEN* is highly expressed during human endoderm differentiation. The depletion of *HIDEN*, both shRNA-mediated knockdown and knockout by deleting the promoter region, severely delays DE differentiation. *HIDEN* is mainly localized in cell cytoplasm and physically interacts with IMP1 protein, which is an important regulator of endoderm differentiation as well. Moreover, the observations that the disruption of *HIDEN* leads to reduced WNT signaling activity, and the impaired DE differentiation due to *HIDEN* or *IMP1* deletion could be restored by WNT signaling activator, synergistically suggest that the WNT signaling pathway acts as the main downstream of *HIDEN*/IMP1 to contribute to endoderm differentiation. In detail, *HIDEN*/IMP1 promotes the mRNA stability of WNT receptor gene *FZD5* through enhancing the interaction of IMP1 and *FZD5* mRNA, and loss of FZD5 significantly blocks endoderm differentiation, together indicating that *FZD5* is a functional target of *HIDEN*/IMP1 in human endoderm differentiation.

LncRNAs have been extensively studied in the last decade, and actively participate in many biological contexts, including pluripotency maintenance and lineage differentiation. However, most current studies of lncRNAs focus on those overlapped lncRNAs and proximal lncRNAs, including divergent lncRNAs, antisense lncRNAs, and enhancer-associated lncRNAs, but desert lncRNAs are often neglected. Here using transcriptome sequencing, RNA-seq following nuclear and cytoplasmic separation during human PSC differentiation, we pay attention to desert lncRNAs and characterize the expression level, subcellular localization, and cell-type specificity. Compared to overlapped lncRNAs and proximal lncRNAs, which are genomically located close to PCGs, desert lncRNAs tend to have higher expression, more cytoplasm-localized, and higher cell-type expression specificity, indicating important biological roles. Thereafter, we have identified a novel desert lncRNA *HIDEN* and revealed the function and mechanism in human endoderm differentiation. As a cytoplasm-localized desert lncRNA, *HIDEN* interacts with IMP1 and together regulate *FZD5* mRNA stability. This illustrated the vital role of lncRNA in a multi-level gene regulation network composed of lncRNA, RNA-binding protein, and signaling pathway, which contributes to the fine-tuned spatial and temporal gene expression programs during lineage specification. It is also noteworthy that *HIDEN* is the first reported functional desert lncRNA in human endoderm differentiation, providing more insights on understanding and efficient acquisition of endoderm-derived functional cells or tissue.

IMP1 is a classical RNA-binding protein and highly associated with tumorigenesis and cancer metastasis [29, 34], but the function of IMP1 in early embryonic development is largely unknown. IMP1 is mainly expressed in the embryonic stage and with negotiable levels in adult tissues [29, 35]. The high expression of IMP1 during embryogenesis indicates a potential role of IMP1 in development and lineage specification. Indeed, IMP1-deficient mice have a smaller size (about 40%) and significant perinatal mortality than normal littermates mainly due to intestinal development defects [36], indicating an important role of IMP1 in endoderm-derived lineage development. A previous report showed that human ESCs with IMP1 knockdown exhibited a reduction in cell adhesion and increase in cell death through its effects on stabilizing *ITGB5* mRNAs and maintaining *BCL2* expression [37]. In our study, we did not observe obvious cell death in either IMP1 knockdown or knockout PSCs; however, we found that the depletion of IMP1 led to an impaired DE differentiation (Fig. 4). Moreover, we revealed that IMP1 interacted with *HIDEN* and together positively regulated WNT signaling pathway by stabilizing *FZD5* mRNA in endoderm differentiation (Figs. 5 and 6). As WNT receptors, frizzled family members such as FZD5, FZD7, and FZD3 could transduce WNT/β-catenin signal to affect human ESC self-renewal and induce differentiation [38]. For example, the disruption of FZD7 impaired the pluripotent state of human ESCs, while selective activation of FZD7 signaling is sufficient to promote mesendodermal differentiation [39]. Here we provided the data that deletion of FZD5 could severely block endoderm differentiation (Fig. 6), together demonstrating the important role of frizzled family in early germ layer differentiation. In addition, these results about loss-of-function of IMP1 (Fig. 4) are consistent with the observation of endodermal intestine defect in IMP1-knockout mice [36], providing a likely mechanism as WNT was critical for endoderm and intestine development [40, 41].

IMP1 binds to massive mRNAs and regulates the turnover during cancer research and embryonic development, but the underlying molecular mechanism, particularly how the target specificity is achieved, is not fully revealed yet [29, 42]. IMP1 contains two RNA-recognition motifs (RRM1, RRM2) in the N-terminal region and four KH domains (KH1-4) in the C-terminal region [29]. However, the in vitro assay indicates the KH domains, rather than RRM domains, of IMP1 are directly responsible for RNA binding, especially KH3 and KH4, which form an intramolecular pseudodimer and create RNA-binding surfaces. More in detail, the highly conserved GXXG loop located in the KH domain is important for RNA binding [43]. The long half-life of IMP-mRNA complexes in vitro supports the notion that the interaction between IMP1 and mRNAs enhances mRNA stability [44, 45]. In our study, *HIDEN* transcript interacts with the KH3-4 domain of IMP1 and enhances *FZD5* mRNA stability through facilitating the interaction of IMP1 protein and *FZD5* mRNA (Fig. 6), which is in accordance with the importance of KH3-4 domains in IMP1 and further indicates the lncRNA-mediated target selectivity of IMP1. Growing evidence indicates that IMP1 binds to mRNAs and together form mRNP granules in cell cytoplasm [45–48], regulating mRNA homeostasis by incorporating its target transcripts into mRNP granules, protecting target mRNAs from miRNA-mediated silencing or releasing them to initiate translation at an appropriate time [49]. In addition, recent studies identified IMP1 as an N6-methyladenosine (m^6^A) reader recognizing the consensus GG(m^6^A)C sequence of target RNAs [44]. More interestingly, the recognition of m^6^A is mediated by the KH3-4 domains of IMP1 [44]. Whether the involvement of *HIDEN* in IMP1-mRNA regulation is related to m^6^A is an interesting question for the following study, and more studies should clarify the structural basis of how lncRNA participates in IMP1-regulated mRNAs stability.

## Conclusions

In summary, we have characterized the biological function of a desert lncRNA *HIDEN* in human endoderm differentiation. *HIDEN* interacts with IMP1 protein and together regulates the stability of *FZD5* mRNA to activate the WNT signal pathway. We further provide functional evidence that IMP1 and FZD5 are required for human DE differentiation. Our findings thus have not only characterized a subset of lncRNAs (i.e., desert lncRNAs), but also revealed the *HIDEN*/IMP1-*FZD5* axis and its important role in human DE differentiation, providing insights in understanding cell fate determination.

## Methods

### Cell culture and differentiation

Two human ESC lines, HUES8 and H9, and one iPSC line PGP1 were used in this study. They were cultured in mTeSR™ medium (STEMCELL Technologies, #05850) on Matrigel-coated plates. Human embryonic kidney 293T (HEK293T) cells were cultured with DMEM medium containing 10% fetal bovine serum (FBS; Gibco, #10270–106) and 1% penicillin–streptomycin (Gibco, #15140163). The endoderm differentiation protocol of human PSCs was based on the previous reports [13, 30] with small adjustments. For HUES8 endoderm differentiation, IMDM (Gibco, #C12440500BT) and F12 (Gibco, #C11765500BT) were mixed at the ratio of 1:1 (IMDM/F12), supplemented with 0.2% BSA (YEASEN, #36106ES76), 1% B27 (without Vitamin A, Shanghai BasalMedia Technologies, S441J7) and 1% penicillin–streptomycin. Activin A (100 ng/ml, PeproTech, #120-14P) was added for 3 or 4 days. For H9 endoderm differentiation, DMEM was used as basal medium instead of IMDM/F12 and the rest ingredients were the same as above. For PGP1 endoderm differentiation, we removed B27 from the medium and treated cells with 1 $\upmu$M JNK-IN-8 (Selleck, S4901) [50] and 100 ng/ml Activin A for day 1, followed by 100 ng/ml Activin A treatment for the next 2 or 3 days. The following pancreatic lineage differentiation was based on the previous report with minor adjustments [27, 51]. Generally, human PSCs were induced into definitive endoderm for four days according to the above method. Then the differentiated cells were cultured in MCDB131 (Gibco, #10372019) supplemented with 0.5% BSA, 1.5% NaHCO_3_, 1% ITS-X (BasalMedia, # S452J7), 1% GlutaMAX (Gibco, #35050061), 1% penicillin–streptomycin, 10 mM Glucose (Invitrogen, #A2494001), 0.25 mM ascorbic acid (Sigma, #A5960), 50 ng/mL KGF (PeproTech, #100–19), 2 $\upmu$M IWR-1 (Selleck, #S7086) for 2 days. Next, cells were treated with MCDB131 supplemented with 2% BSA, 2.5% NaHCO_3_, 1% ITS-X, 1% GlutaMAX, 1% penicillin–streptomycin, 10 mM Glucose, 0.25 mM Vitamin C, 50 ng/ mL KGF, 2 $\upmu$M IWR-1, 0.25 $\upmu$M SANT1 (Selleck, #S7092), 200 nM LDN193189 (Selleck, #S7507), 100 nM TTNPB (Selleck, #S4627) and 500 nM PDBU (Sigma, #P1269) for 4 days (pancreatic progenitors 1, PP1 stage). During pancreatic progenitors 2 stage (PP2), 400 nM LDN193189, 10 nM TTNPB and 250 nM PDBU were used, and the rest gradients were the same as PP1 stage for 4 days. For EB differentiation, human PSCs were digested into single cells by Accutase and then counted and resuspended at the density of 200 cells/$\upmu$L in mTeSR1 with 10 μM Y-27632. Then the single cell drops (10 μL) were hanging on the lid of Petri dishes. The aggregated EBs were collected into 6-well low attachment plates after one day culture. EB medium (DMEM, 10% FBS, 1% penicillin–streptomycin) was changed every two days. After 9 days’ spontaneous differentiation, the differentiated EBs were harvested for qPCR assay. Other chemicals used in this study included CHIR-99021 (also CHIR, Selleck, #S2924), Wnt-C59 (Selleck, #S7037).

### Plasmid constructs of shRNA knockdown

The shRNAs specifically against *HIDEN*, *IMP1*, *FZD5* and scramble control were cloned into lentiviral vector pTY plasmid. HEK293T were transfected with lentiviral plasmid expressing shRNA or scramble control shRNA (shC) and lentiviral helping vectors (pNHP, pCEP-TAT, pHEF-VSVG) for lentivirus packaging. The stable PSC lines were established by selection with 2 $\upmu$g/mL puromycin for two weeks. The targeting sequences of the effective shRNAs were provided in Additional file 9: Table S7.

### CRISPR-Cas9 mediated knockout

Genome editing was performed by electroporating human PSC cells with pX459 plasmid expressing target sgRNAs. Cells were selected with 2 $\upmu$g/mL puromycin for 2 days, followed by single cell sorting (BD FACS Aria) and genotyping. The sequences of all sgRNAs and the primers for genomic sequencing were listed in Additional file 9: Table S7.

### RACE and cDNA cloning

After 4 days of endoderm differentiations, HUES8 cells were harvested and total RNA was isolated using Hipure Total RNA Mini Kit (Magen, #R4111-03). The 5’ and 3’ fragments of *HIDEN* were amplified using SMARTer RACE 5'/3' Kit (TAKARA, #634858) according to the manufacturer's instructions, then PCR products were cloned for Sanger sequencing. The gene-specific primers and primers used to clone full length of *HIDEN* were listed in Additional file 9: Table S7. The full length of *HIDEN* transcript was shown in Additional file 3.

### Cell proliferation assay

Human PSCs were treated with Accutase, then counted and seeded in 96-well plates coated with Matrigel at a density of 3000 cells/well followed by culturing for 48, 72, or 96 h. Before detection, the plates were replenished with fresh medium containing 10 μl CCK for each well and incubated for 4 h at 37 °C according to the manufacturer's instructions. After shaking on an orbital shaker, the optical density (OD) was measured at 450 nm with MD SpectraMax i3x.

### Quantitative RT-qPCR

Total RNA was isolated from cultured cells using Hipure Total RNA Mini Kit (Magen) or TriPure isolation reagent (Roche, #11667165001) according to the manufacturer's instructions. First strand cDNA was synthesized by reverse transcription of 1 $\upmu$g RNA using ABScript II RT Master MIX (ABclonal, #RK20402). 2 $\times$ SYBR Green qPCR Master Mix (Bimake, #B21203) was used for quantification of gene expression on a CFX384 qPCR machine (Bio-Rad). *GAPDH* served as the internal control for normalization. One-tail unpaired *t*-test was performed to obtain p-values for RT-qPCR experiments. The primers used in all qPCR assays were listed in Additional file 9: Table S7.

### Flow cytometric analysis

Differentiated PSCs were digested into single cells by TrypLE (Gibco, #12604021) and washed twice with DPBS containing 2% FBS. Cells were then incubated with CD184-APC (BD, #555976) for 30 min. As for the following intracellular flow cytometry, cells were fixed according to the manufacturer's instructions of Transcription Factor Buffer Set (BD, #562574), and then incubated with SOX17-Alexa488 (BD, #562205) antibody. Corresponding isotype was used as control. The SOX17^+^ or CXCR4^+^ cells were detected by FACSCelesta flow cytometer (BD) or CytoFLEX flow cytometer (Beckman Coulter) and analyzed by FlowJo software.

### Western blot

Cells were lysed in RIPA buffer (Beyotime, #P0013C) with cocktail (Roche, #4693132001). The lysates were separated by 10% SDS-PAGE and immunoblotted with indicated antibodies. The primary antibodies used in this study included: SOX17 (R&D, #AF1924, 1:1000), FOXA2 (R&D, #AF2400, 1:1000), IGF2BP1 (IMP1, ABclonal, #A1517, 1:1000), IGF2BP2 (IMP2, ABclonal, #A2749, 1:1000), IGF2BP3 (IMP3, ABclonal, #A4444, 1:1000), GAPDH (Proteintech, #10494-1-AP, 1:5000), H3 (Proteintech, #17,168-1-AP, 1:3000), active β-catenin (CST, #8814, 1:1000), β-catenin (CST, #8480, 1:1000). After the overnight incubation of primary antibodies at 4 °C, the membrane was washed and incubated with secondary antibodies at room temperature for 2 h. The proteins were visualized by the ECL detection reagents (Millipore, #WBUSLS0100).

### Immunofluorescence staining

Cells were fixed in 4% paraformaldehyde after PBS washing, then blocked and permeabilizated by blocking buffer (PBS with 10% donkey serum and 0.3% Triton X-100). Cells were incubated overnight at 4 °C by blocking buffer with the primary antibodies at proper concentration. The used primary antibodies in the experiment were: SOX17 (R&D, #AF1924, 1:200), FOXA2 (R&D, #AF2400, 1:200), OCT4 (CST, #2750, 1:200), SOX2 (BD, #561,469, 1:200), NANOG (CST, #4903, 1:200), SSEA4 (Millipore, #MAB4304, 1:400), TRA-1-60 (Millipore, #MAB4360, 1:400), PDX1 (R&D, #AF2419, 1:200). After washing with PBS for three times, cells were incubated with blocking buffer with the corresponding secondary fluorescent antibodies. The results of immunofluorescence were visualized and imaged under an Olympus IX53 microscope.

### Cytosolic/nuclear fractionation

Differentiated endoderm cells were grown on 6-well plates and harvested at day 4. Cells were lysed in CE buffer (10 mM Hepes, 60 mM KCl, 1 mM EDTA, 0.34 M sucrose, 0.3% NP-40, 1 mM DTT) with cocktails for 5 min on ice, then centrifugated at 3000 rpm for 15 min to separate nuclear and cytoplasmic compartments. TriPure isolation reagent (Roche, #11667165001) was used to extract nuclear or cytoplasmic RNA, followed by RT-qPCR analysis. The *GAPDH*, *ACTB* and *SOX17* mRNAs were used as cytoplasmic controls, while lncRNA *MALAT1* and *NEAT1* were used as nuclear controls.

### RNA immunoprecipitation (RIP)

RIP was performed with the Magna RNA-binding protein immunoprecipitation kit (Millipore, #17–700) following the manufacturer's instructions. Cells lysis from 1 $\times$ 10^7^ cells and 6 μg IMP1 or IgG antibody were used. RNA was isolated with TRIzol (Invitrogen, #10296010) and phenol/chloroform/isoamyl alcohol and then analyzed by qRT-PCR.

### RNA pull-down

For RNA pull-down assay, full-length *HIDEN* and its antisense and other control RNAs were transcribed in vitro with HiScribe T7 High Yield RNA Synthesis Kit (NEB, #E2040S) and biotin-16-UTP (Roche, #11388908910) according to the manufacturer's instructions. 10 μg biotin-labeled *HIDEN* or control RNAs was incubated with total cell lysate of differentiated endoderm cells from PSCs or HEK293T cells. Then 40 μL Dynabeads MyOne Streptavidin C1 (Invitrogen, #65001) were added to isolate the RNA-protein complex, followed by silver staining / mass spectra or Western blot. RNA pulled down by RNA were conducted as above, then digested by proteinase K at 55 °C incubator and extracted by phenol chloroform.

### RNA sequencing and RIP-sequencing data analysis

For RNA-sequencing, total RNA was isolated with HiPure Total RNA Mini Kit. RNA libraries were sequenced on an Illumina Hiseq X Ten platform with paired-end reads at Geekgene Technology. RNA-seq raw data that contained adapters were removed and trimmed by Trim Galore (v0.6.6). The clean data were aligned to human GRCh38 genome reference with the HISAT2 [52]. The GENCODE V29 gene transfer format (GTF) was used to count reads by the FeatureCounts (v2.0.1). All counts were further normalized with TPM (Transcripts Per Million) in R software. Differential expression analysis was performed for binary comparisons using the R package DESeq2 [53]. For cutoff threshold, we set *P* value < 0.05, abs (log_2_(fold-change)) > 1.0 in *HIDEN*-KO dataset and abs (log_2_ (fold-change)) > 0.5 in *IMP1*-KO dataset separately. Gene Ontology analysis was executed by R package clusterProfile [54].

To investigate the expression level of different kinds of lncRNAs during endoderm differentiation, we reanalyzed three RNAseq datasets in different PSC lines and the DE derivates, including H9 dataset from GSE44875 [12], HUES8 dataset from GSE137208 [55] and GSE143499 [13], PGP1 dataset from GSE173690 [56] and GSE188501 [57]. The subcellular localization of lncRNAs were calculated using the “relative concentration index” (RCI) with slight modification [19]. The expression level of different components was normalized as FPKM and the RCI was calculated as below: CN.RCI = log_2_(CE/NE) + log_2_(CE/NM) (CE: cytoplasmic elution component, NE: nuclear elution component, NM: nuclear insoluble component). In addition, we reanalyzed the H1 and A549 datasets from GSE30567 [58] to verify the results. To quantify the stage- or cell-specificity of PCGs and lncRNAs expression, we calculated the specificity score in two different manners according to the references [12, 21]. The dataset from GSE44875 [12] and GSE134743 [59] was reanalyzed by the same pipeline. The coding potential of gencode.v29. transcripts were predicted using the software GeneID (v1.4.5) (https://ftp.ebi.ac.uk/pub/databases/gencode/Gencode_human/release_29/gencode.v29.transcripts.fa.gz) with the parameters in file human.070123.param, and additional -s -3 parameters [21, 60]. To investigate the expression level of *HIDEN* in human tissues, we downloaded and reanalyzed the 22,952 tissues expression data including 30 human tissues (https://www.gtexportal.org/). The gene expression data (TPMs) (https://storage.googleapis.com/gtex_analysis_v8/rna_seq_data/GTEx_Analysis_2017-06-05_v8_RNASeQCv1.1.9_gene_tpm.gct.gz) and the sample annotation file (https://storage.googleapis.com/gtex_analysis_v8/annotations/GTEx_Analysis_v8_Annotations_SampleAttributesDS.txt) were integrated and further analyzed in R software.

For IMP1-RIP-seq, the raw data that contained adapters were removed and trimmed by Trim Galore (v0.6.6) and then aligned to human GRCh38 genome reference with the HISAT2. To identify differential peaks between Input and IP group, MACS2 (v2.1.1) was used with default parameters in IMP1-RIPseq, which uses a dynamic Poisson distribution to effectively capture local biases in the genome [61–63], allowing for more robust predictions. To find IMP1 reduced enrichment genes in *HIDEN*-KO, we compared IMP1-RIPseq peaks in wild type and *HIDEN*-KO group using macs2 bdgdiff (a subcommand of MACS2). Then the peaks were annotated to genes by R package ChIPseeker [64]. The peak visualization was performed in IGV (v2.6.2). In order to make the data comparison more obvious, we overlaid the IP track to input track in IGV and subtracted input signal from IP signal by bigwigCompare command of deepTools [65] respectively (Fig. 6e).

### Protein expression and purification

IMP1 and its mutants were inserted into pET28a plasmid containing His tag. The fusion proteins were expressed in *E. coli* strain Rosetta (Shanghai Weidi Biotechnology, #EC1010) under 0.5 mM IPTG, 25 °C for 4-6 h induction after grown to OD_600_ 0.6-0.8. After IPTG induction, *E. coli* cell pellets were collected, then resuspended with His Lysis buffer (50 mM Tris-HCl pH8.0, 500 mM NaCl, 10% glycerol, 1.0% Triton X-100, 10 mM Imidazol, 0.2 mM PMSF), and further crushed using high pressure homogenizer. Protein supernatant was collected by centrifugation at 12,500 rpm for 15 min at 4 °C. About 150-200 $\mathrm{\mu L}$ Ni-NTA Agarose beads (Abclonal, #AS045) were washed twice using His Lysis buffer and then incubated with protein supernatant at 4 °C for 3-4 h. The beads were collected and washed twice for 10 min using 5 mL His Washing buffer (50 mM Tris-HCl pH 8.0, 500 mM NaCl, 10% glycerol, 20 mM Imidazol). Finally, the protein was eluted from the beads using 500 $\mathrm{\mu L}$ His Elution buffer (20 mM Tris-HCl pH 8.0, 500 mM NaCl, 10% glycerol, 300 mM Imidazol). The eluted protein was concentrated using Amicon Ultracentrifuge filters (Merck, #UFC201024). Protein purity and concentration were determined with SDS-PAGE followed by Coomassie blue staining. BSA was used as protein standard sample.

### EMSA (electrophoretic mobility shift assay)

EMSA was performed as reference [15] with slight modifications. Briefly, RNA was labelled with Cy5-UTP (APExBIO, #B8333) using HiScribe T7 High Yield RNA Synthesis Kit (NEB, #E2040S). The 40 nM Cy5-labelled RNA probes were denatured by heating at 95 °C for 1 min and cooling on ice, and then adding an equal volume of 2 $\times$ EMSA binding buffer (40 mM Tris-HCl pH 7.9, 20% glycerol, 300 mM KCl, 5 mM MgCl_2_, 2 mM DTT, 0.2 mg/ml BSA and 10 U of RNase inhibitor) and incubated at room temperature for 30 min. Purified IMP1 or mutant proteins was added and then incubated with RNA at room temperature for 30 min. After incubation, the RNA-protein complexes were separated and analyzed using 0.5% native agarose gel. The images were captured using Biorad ChemiDoc MP.

### Statistical analysis

Statistical analysis was conducted using PRISM 8.0 software. The results were shown as means $\pm$ SD from at least three independent experiments. Single comparison between two groups was analyzed by two-tailed unpaired *t*-test. Comparisons between multiple groups were determined using One-Way ANOVA analysis. Pearson's correlation analysis was used to evaluate the correlation between two variables. *P* value < 0.05 is considered statistically significant (* means *p* < 0.05, ** means *p* < 0.01, and *** means *p* < 0.001), while “n.s.” stands for not statistically significant.

## Supplementary Information

Additional file 1: **Fig. S1.** Desert lncRNAs are highly expressed during human endoderm differentiation. **Fig. S2.** *HIDEN* knockout is not essential for PSC pluripotency but required for endoderm lineage differentiation. **Fig. S3.** *HIDEN* knockdown exhibits decreased endoderm differentiation. **Fig. S4.** *HIDEN* physically interacts with IMP1. **Fig. S5.** IMP1 is required for DE differentiation. **Fig. S6.** WNT pathway is regulated by *HIDEN*/IMP1. **Fig. S7.** *HIDEN* promotes WNT pathway through FZD5.

Additional file 2: **Table S1.** ESC and DE differentially expressed lncRNAs in three datasets. ESC and DE differentially expressed deserted lncRNAs in H9 cells described in Figure 1f.

Additional file 3: *HIDEN* sequence.

Additional file 4: **Table S2.** The transcript per millionvalue of gene expression in wildtype or *HIDEN*-KO DE cells. The transcript per millionvalue of gene expression in wildtype or IMP1-KO DE cells.

Additional file 5: **Table S3.** *HIDEN*-KO upregulated or downregulated GO terms. IMP1-KO downregulated GO terms. GO terms of the overlap between *HIDEN*-KO DEGs and IMP1 RIP-seq enriched genes. GO analysis of *HIDEN*-KO reduced genes.

Additional file 6: **Table S4.** The mass spectrometry results of specific 72 KD band and whole IP extracts.

Additional file 7: **Table S5.** IMP1-bound genes identified by IMP1 RIP-seq. The IMP1-enriched genes whose binding with IMP1 was reduced by *HIDEN*-KO.

Additional file 8: **Table S6.** WNT associated gene list.

Additional file 9: **Table S7.** Oligos used in this study. shRNA target sequences, CRISPR sgRNA target sequence, primers used in genomic genotyping, gene-specific primers used in RACE assays and full-length cloning, and qPCR primers were shown.

Additional file 10: **Table S8.** GEO information used in this study.

Additional file 11: Uncropped images for western blots in this manuscript.

Additional file 12: Review history.

## Data Availability

Three RNA-seq datasets in different human PSC lines and the DE derivates, including H9 dataset from GSE44875 [12], HUES8 dataset from GSE137208 [55] and GSE143499 [13], PGP1 dataset from GSE173690 [56] and GSE188501 [57], H1 and A549 datasets from GSE30567 [58], other cell-types (ARPE-19–1, H1, H9, HepG2 and Jurkat) dataset from GSE134743 [59] were used in this study, the detailed accession numbers for each sample are listed in Additional file 10: Table S8. The RNA-seq, RIP-seq data generated in this study are available under accession number of GSE188501 [57].
