# Supplementary material for: A desert lncRNA HIDEN regulates human endoderm differentiation via interacting with IMP1 and stabilizing FZD5 mRNA
Source: Genome Biol. 2023 Apr 24;24:92. doi: 10.1186/s13059-023-02925-w (PMC10124006; doi:10.1186/s13059-023-02925-w)
Supplement: Supplementary file 1 — Fig. S1. Desert lncRNAs are highly expressed during human endoderm differentiation. Fig. S2. HIDEN knockout is not essential for PSC pluripotency but required for endoderm lineage differentiation. Fig. S3. HIDEN knockdown exhibits decreased endoderm differentiation. Fig. S4. HIDEN physically interacts with IMP1. Fig. S5. IMP1 is required for DE differentiation. Fig. S6. WNT pathway is regulated by HIDEN/IMP1. Fig. S7. HIDEN promotes WNT pathway through FZD5. [file 13059_2023_2925_MOESM1_ESM.pdf]

Figure S1

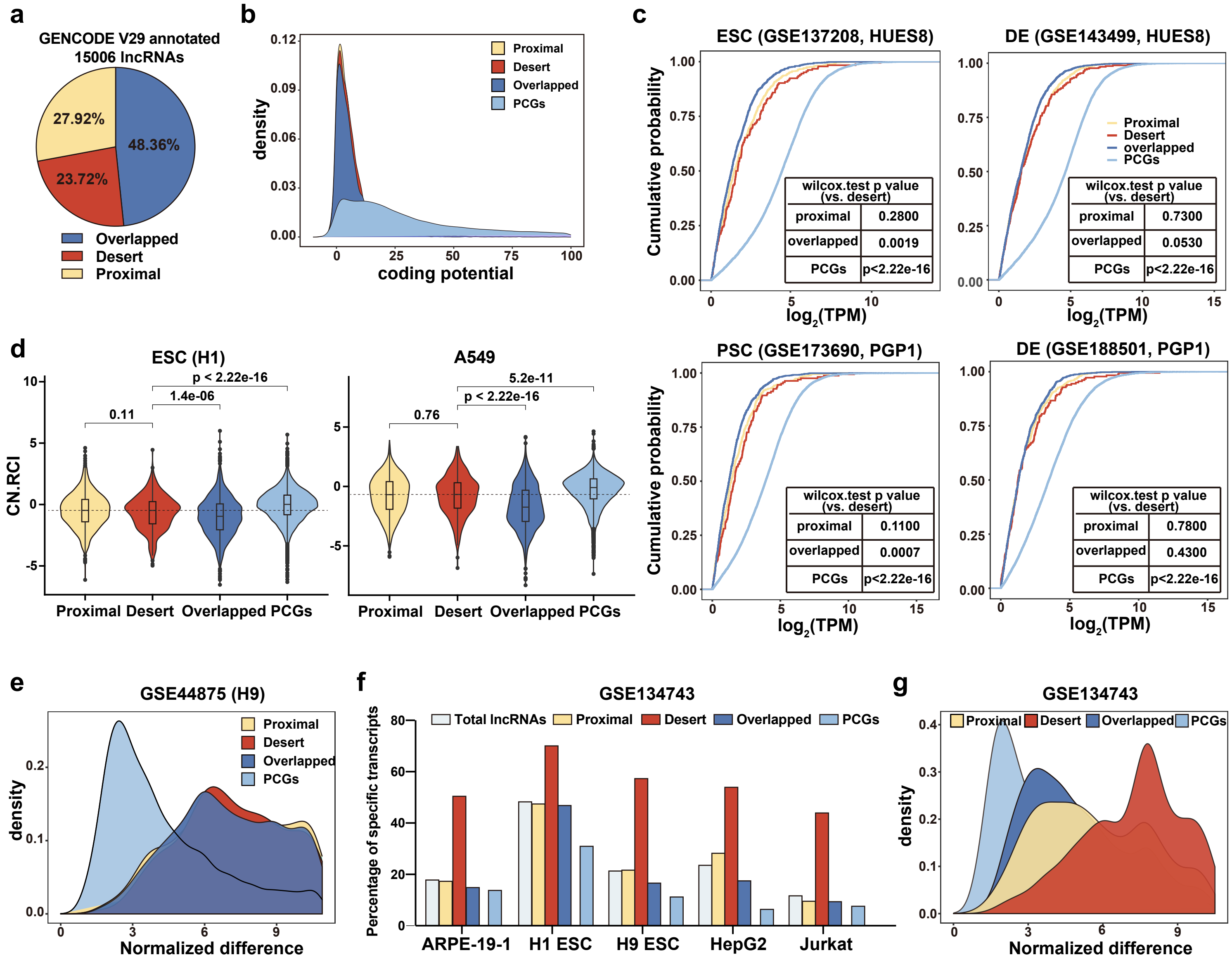

h

Software	Sequence	RNA size	ORF size	Ficket Score	Hexamer Score	Coding Probability	Coding Label	Website
CPAT	HIDEN	1045	237	0.4923	-0.0005937	0.01704	noncoding	http://lilab.research.bcm.edu/cpat/

Software	Sequence	RNA size	ORF size	Ficket Score	ORF integrity	Coding Probability	Coding Label	Website
CPC2	HIDEN	1045	79	0.3653	complete	0.08962	noncoding	http://cpc2.gao-lab.org/

Figure S1. Desert lncRNAs are highly expressed during human endoderm differentiation.

(**a**) The percentage of overlapped, proximal, and desert lncRNAs in GENCODE V29 annotated 15006 lncRNAs.

(**b**) The predicted coding potential of lncRNAs and PCGs.

(**c**) The expression level of differentially expressed lncRNA subsets and PCGs from transcriptome analysis of PSCs and DE cells. The p value between desert lncRNAs and other subsets were listed in the chart.

(**d**) The subcellular localization of lncRNAs and PCGs, calculated by "relative concentration index" (RCI) based on the transcriptome analysis following cellular compartment separation in H1 ESC and human lung cancer cell A549.

(**e**) The cell expression specificity of lncRNAs and PCGs in ESCs, DE, pancreatic endocrine (PP), pancreatic alpha and beta cells indicated by Normalized Difference.

(**f-g**) The cell expression specificity of lncRNAs and PCGs in ESCs and other cell lines such as ARPE-19-1 (human retinal pigment epithelia cell line), HepG2 (liver hepatocellular cancer cell line), Jurkat (immortalized human T lymphocyte cell line), calculated by specificity score (**f**) or Normalized Difference (**g**). The GEO accession number was indicated.

(**h**) The protein-coding potential of *HIDEN* was predicted on CPAT and CPC2 online tools.

Figure S2

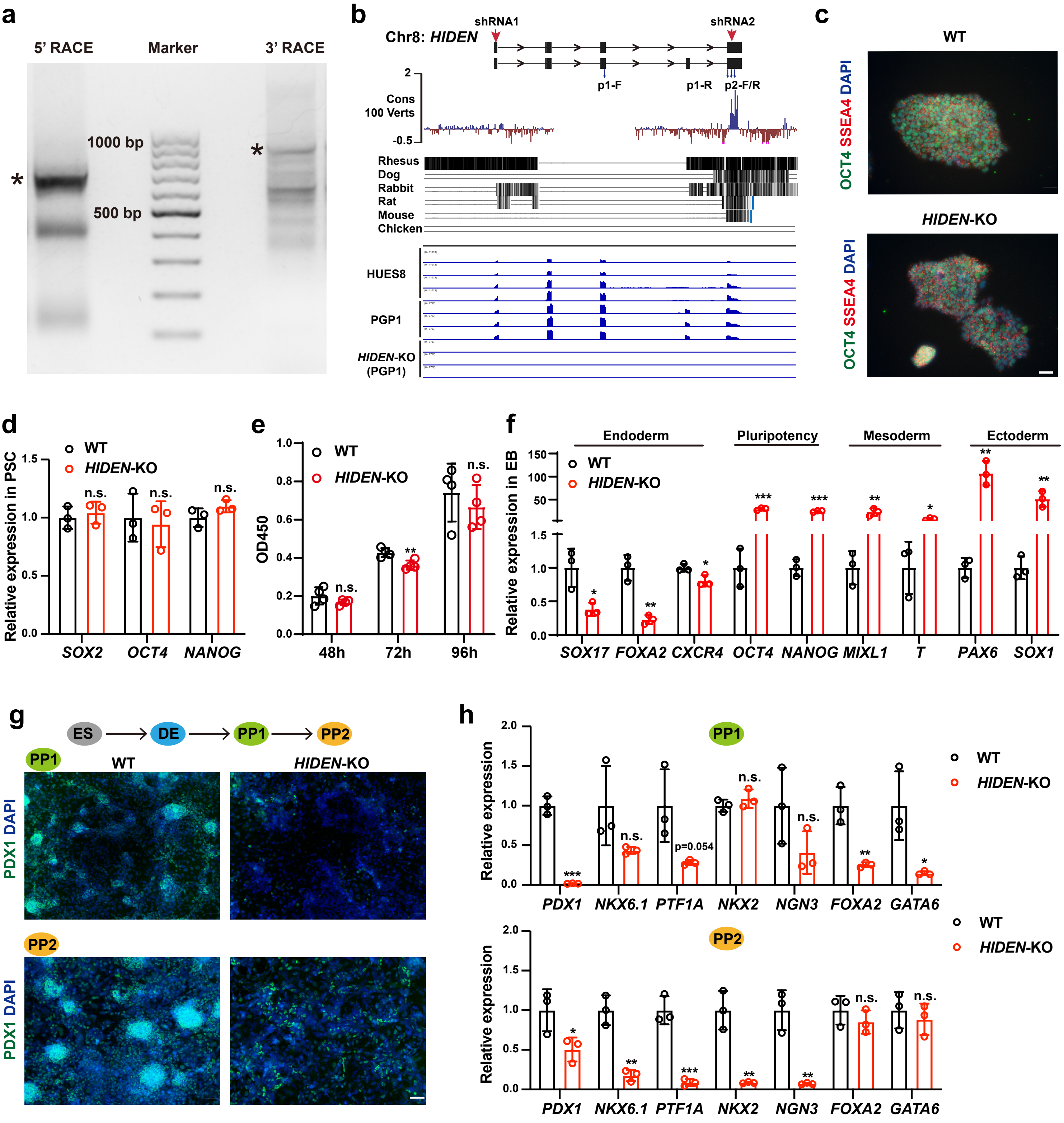

a
5' RACE
Marker
3' RACE
1000 bp
500 bp
b
Chr8: HIDEN
shRNA1
shRNA2
p1-F
p1-R
p2-F/R
Cons 100 Verts
Rhesus
Dog
Rabbit
Rat
Mouse
Chicken
HUES8
PGP1
HIDEN-KO (PGP1)
c
WT
HIDEN-KO
OCT4 SSEA4 DAPI
d
WT
HIDEN-KO
Relative expression in PSC
SOX2
OCT4
NANOG
n.s.
e
OD450
48h
72h
96h
n.s.
f
Endoderm
Pluripotency
Mesoderm
Ectoderm
Relative expression in EB
SOX17
FOXA2
CXCR4
OCT4
NANOG
MIXL1
T
PAX6
SOX1
g
ES
DE
PP1
PP2
PDX1 DAPI
h
Relative expression
PDX1
NKX6.1
PTF1A
NKX2
NGN3
FOXA2
GATA6
p=0.054

Figure S2. *HIDEN* knockout is not essential for PSC pluripotency but required for endoderm lineage differentiation.

(**a**) The 5' and 3' ends of the *HIDEN* transcript were defined by RACE-PCR. "*" indicated that the RACE fragment and the lower bands were proved to be non-specific amplification through DNA sequencing.

(**b**) Top: the full length of *HIDEN* was cloned from the cDNA of HUES8 DE cells. PhyloP value of Vertebrates basewise conservation on *HIDEN* genomic region was shown. *HIDEN* shRNA target sites and qPCR primers were indicated by red arrows and blue arrows respectively. Bottom: the RNA-seq coverage plot of *HIDEN* in HUES8 DE cells, PGP1 DE cells and *HIDEN*-KO DE cells.

(**c**) Immunofluorescent staining of pluripotency markers (OCT4, SSEA4) in wildtype and *HIDEN* knockout PSCs. Scale bar = 50 μm.

(**d**) qPCR analysis of representative pluripotency genes in wildtype and *HIDEN* knockout PSCs (n=3).

(**e**) Cell proliferation detected by CCK kit at different time points in wildtype and *HIDEN* knockout PSCs (n=4).

(**f**) RNA expression of representative endoderm genes, pluripotency genes, mesoderm genes and ectoderm genes were examined in wildtype or *HIDEN*-KO EB (n=3).

(**g-h**) The immunofluorescence staining results of pancreatic marker PDX1 (**g**) and RNA expression levels of pancreatic transcription factors (**h**) in wildtype and *HIDEN* knockout cells at PP1 (pancreatic progenitors 1) and PP2 (pancreatic progenitors 2) stage (n=3). Scale bar = 100 μm.

Figure S3

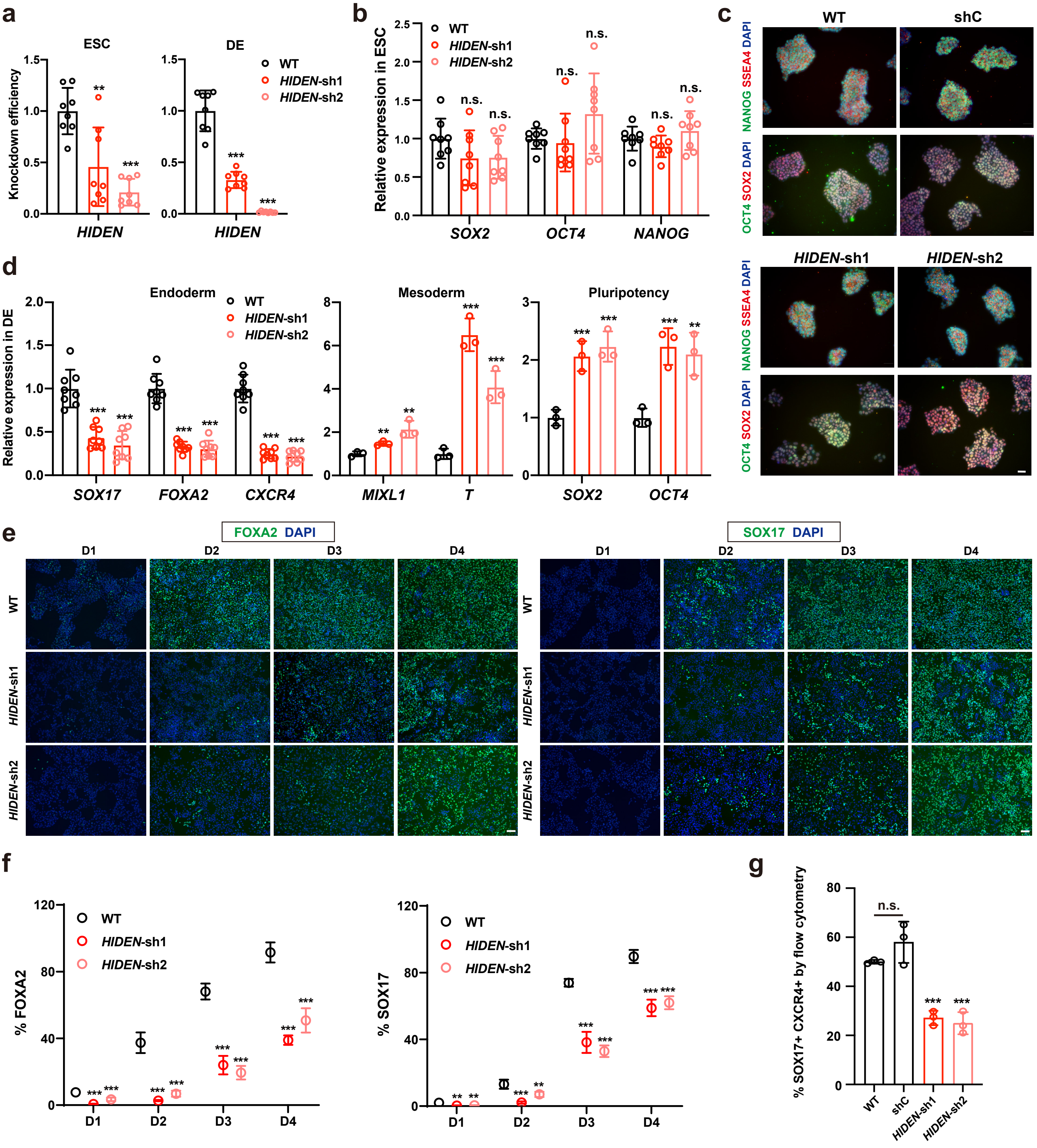

a
ESC
DE
Knockdown efficiency
WT
HIDEN-sh1
HIDEN-sh2
HIDEN
b
Relative expression in ESC
n.s.
SOX2
OCT4
NANOG
c
WT
shC
HIDEN-sh1
HIDEN-sh2
OCT4 NANOG SSEA4 DAPI
OCT4 SOX2 DAPI
d
Endoderm
Mesoderm
Pluripotency
Relative expression in DE
SOX17
FOXA2
CXCR4
MIXL1
T
SOX2
OCT4
e
FOXA2 DAPI
SOX17 DAPI
D1
D2
D3
D4
WT
HIDEN-sh1
HIDEN-sh2
f
% FOXA2
% SOX17
g
% SOX17+ CXCR4+ by flow cytometry
WT
shC
HIDEN-sh1
HIDEN-sh2

Figure S3. *HIDEN* knockdown exhibits decreased endoderm differentiation.

(**a**) *HIDEN* KD efficiencies were examined by RT-qPCR in wildtype and *HIDEN*-KD ESCs and DE cells (n=8).

(**b**) The expression levels of pluripotency genes were examined in wildtype or *HIDEN* knockdown ESCs (n=8).

(**c**) Immunofluorescence staining results of pluripotency genes (OCT4, SOX2, NANOG, SSEA4) in wildtype, shRNA control (shC) or *HIDEN* knockdown ESCs. Scale bar = 50 μm.

(**d**) RNA expression levels of marker genes, including endoderm genes (n=8), mesoderm genes (n=3), and pluripotency genes (n=3), were examined in wildtype or *HIDEN* knockdown DE cells.

(**e-f**) The immunofluorescence staining results of endoderm markers FOXA2 and SOX17 in wildtype and *HIDEN* knockdown DE cells during four days of endoderm differentiation. Scale bar = 100 μm. Quantitative results were shown (**f**) (n=5).

(**g**) The statistical results of flow cytometric analysis of the SOX17 and CXCR4 positive cells in wildtype, shRNA control and *HIDEN* knockdown DE cells (n=3).

Figure S4

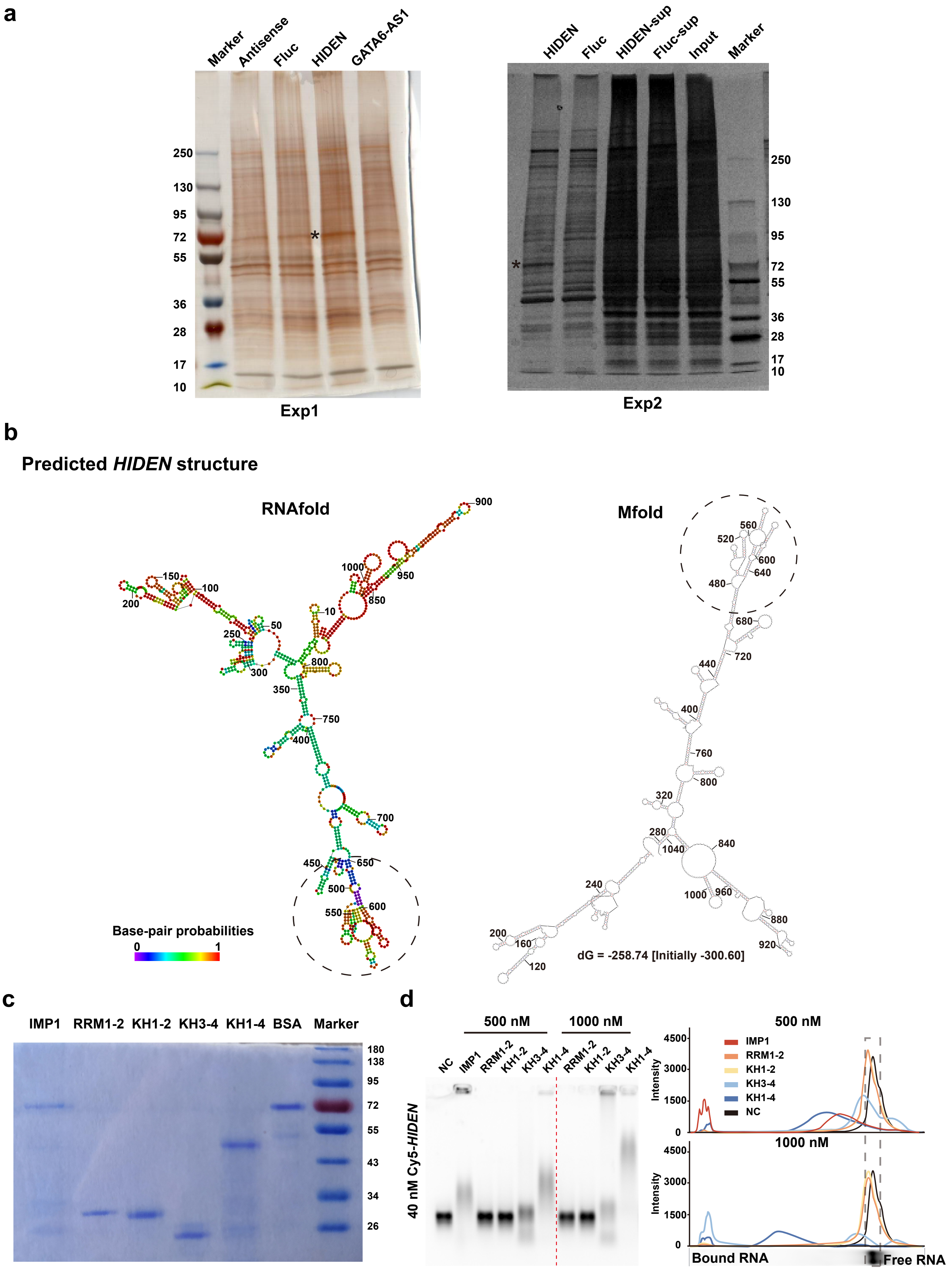
a
Marker
Antisense
Fluc
HIDEN
GATA6-AS1
250
130
95
72
55
36
28
17
10
*
Exp1
HIDEN
Fluc
HIDEN-sup
Fluc-sup
Input
Marker
250
130
95
72
55
36
28
17
10
*
Exp2
b
Predicted HIDEN structure
RNAfold
Mfold
Base-pair probabilities
0
1
dG = -258.74 [Initially -300.60]
c
IMP1
RRM1-2
KH1-2
KH3-4
KH1-4
BSA
Marker
180
138
95
72
55
43
34
26
d
500 nM
1000 nM
NC
IMP1
RRM1-2
KH1-2
KH3-4
KH1-4
40 nM Cy5-HIDEN
500 nM
1000 nM
Intensity
4500
3000
1500
0
IMP1
RRM1-2
KH1-2
KH3-4
KH1-4
NC
Bound RNA
Free RNA

Figure S4. *HIDEN* physically interacts with IMP1.

(**a**) The silver staining results of RNA pulldown in DE cells. Antisense *HIDEN*, *Luciferase* (*Fluc)* and unrelated lncRNA *GATA6-AS1* were used as negative controls. "*" indicated the specific band around 72 kDa in *HIDEN*-IP extracts. Two independent experiments were shown.

(**b**) The predicted RNA secondary structure of *HIDEN* using RNAfold and Mfold.

(**c**) Coomassie brilliant blue staining results of purified His-tagged IMP1 and IMP1 truncations expressed in *E. coli*. BSA was used to normalize protein concentration.

(**d**) EMSA results of *in vitro* transcribed Cy5-labeled *HIDEN* transcript (40 nM) and IMP1 truncated proteins at different concentrations.

Figure S5

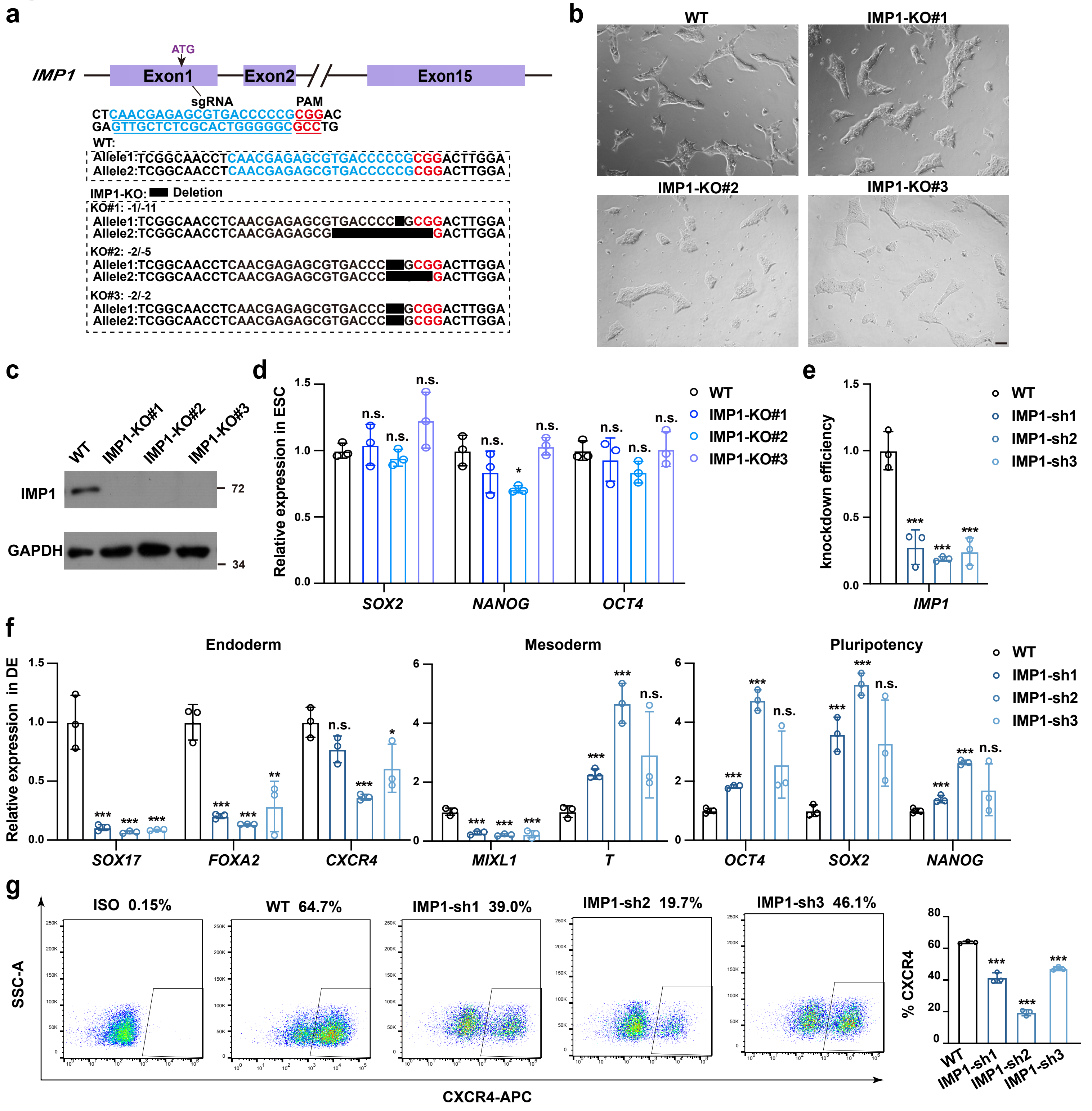
a
IMP1
ATG
Exon1
Exon2
Exon15
sgRNA
PAM
CTCAACGAGAGCGTGACCCCCGCGGAC
GAGTTGCTCTCGCACTGGGGGCGCCTG
WT:
Allele1:TCGGCAACCTCAACGAGAGCGTGACCCCCGCGGACTTGGA
Allele2:TCGGCAACCTCAACGAGAGCGTGACCCCCGCGGACTTGGA
IMP1-KO: Deletion
KO#1: -1/-11
KO#2: -2/-5
KO#3: -2/-2
b
WT
IMP1-KO#1
IMP1-KO#2
IMP1-KO#3
c
IMP1
GAPDH
72
34
d
Relative expression in ESC
SOX2
NANOG
OCT4
n.s.
e
knockdown efficiency
IMP1
IMP1-sh1
IMP1-sh2
IMP1-sh3
f
Endoderm
Mesoderm
Pluripotency
Relative expression in DE
SOX17
FOXA2
CXCR4
MIXL1
T
OCT4
SOX2
NANOG
g
ISO 0.15%
WT 64.7%
IMP1-sh1 39.0%
IMP1-sh2 19.7%
IMP1-sh3 46.1%
SSC-A
CXCR4-APC
% CXCR4

Figure S5. IMP1 is required for DE differentiation.

(**a**) The genotypes of the three IMP1 knockout H9 ESC lines. sgRNA target sequence and PAM sequence were indicated in blue and red respectively. Deleted nucleotides were marked in black squares.

(**b**) The bright field image of IMP1 knockout ESCs. Scale bar=200 μm.

(**c**) IMP1 knockout detected by immune blot.

(**d**) The pluripotency gene expression in wildtype and IMP1 knockout ESCs (n=3).

(**e**) The knockdown efficiency of IMP1 in three IMP1-KD ESC cell lines (n=3).

(**f**) RT-qPCR analysis of representative endoderm genes, mesoderm genes and pluripotency genes in wildtype and IMP1-knockdown DE cells (n=3).

(**g**) Flow cytometric analysis of $CXCR4^+$ cells in wildtype and IMP1-knockdown DE cells (n=3).

Figure S6

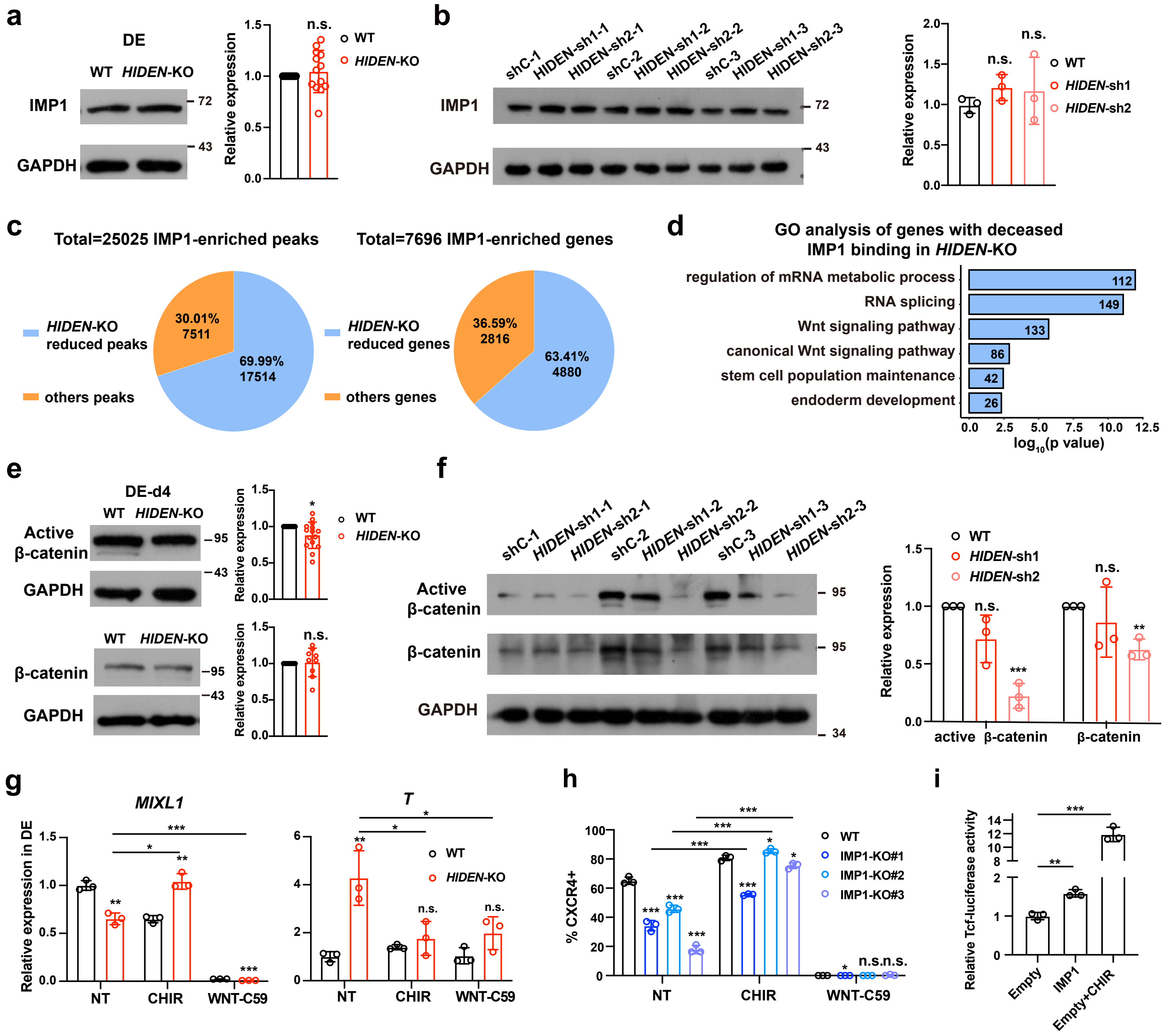
a
DE
WT HIDEN-KO
IMP1
GAPDH
b
IMP1
GAPDH
c
Total=25025 IMP1-enriched peaks
Total=7696 IMP1-enriched genes
HIDEN-KO reduced peaks
others peaks
HIDEN-KO reduced genes
others genes
d
GO analysis of genes with deceased IMP1 binding in HIDEN-KO
regulation of mRNA metabolic process
RNA splicing
Wnt signaling pathway
canonical Wnt signaling pathway
stem cell population maintenance
endoderm development
e
DE-d4
Active β-catenin
β-catenin
GAPDH
f
Active β-catenin
β-catenin
GAPDH
g
MIXL1
T
h
% CXCR4+
i
Relative Tcf-luciferase activity
Empty
IMP1
Empty+CHIR

Figure S6. WNT pathway is regulated by *HIDEN*/IMP1.

(**a**) The protein level of IMP1 in wildtype and *HIDEN* knockout DE cells (n=13).

(**b**) The protein level of IMP1 in wildtype and *HIDEN* knockdown DE cells (n=3).

(**c**) IMP1-bound peaks or genes decreased by *HIDEN*-KO, determined by RIP-seq in wildtype and *HIDEN*-KO DE cells were shown.

(**d**) The GO analysis of genes with decreased IMP1 binding in *HIDEN*-KO cells.

(**e**) The protein level of active β-catenin (n=15) and total β-catenin (n=9) in wildtype and *HIDEN* knockout DE cells.

(**f**) The protein level of active β-catenin and total β-catenin in wildtype and *HIDEN* knockdown DE cells (n=3).

(**g**) RT-qPCR analysis of mesoderm genes (*MIXL1*, *T*) in wildtype or *HIDEN*-knockout DE cells treated with WNT signaling activator (CHIR at 1 μM) or inhibitor (Wnt-C59 at 1 μM) (n=3).

(**h**) Flow cytometry analysis of $CXCR4^+$ cells in wildtype or IMP1-knockout DE cells treated with WNT signaling activator (CHIR at 1 μM) or inhibitor (Wnt-C59 at 1 μM) (n=3).

(**i**) The TCF-luciferase activity in 293T transfected with IMP1 or empty vector (n=3). Cells treated with 1 μM CHIR were used as positive controls.

Figure S7

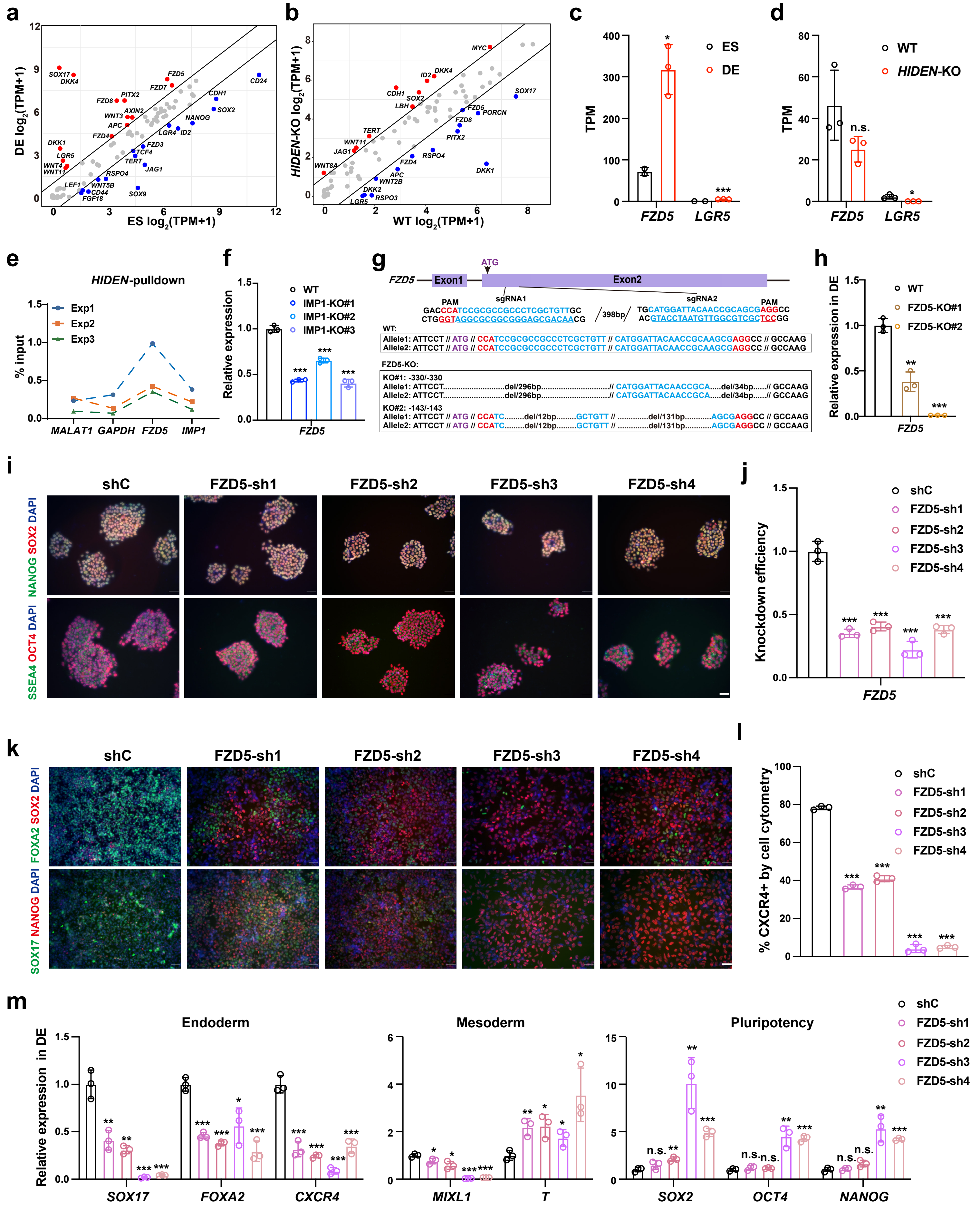
a
DE log2(TPM+1)
ES log2(TPM+1)
SOX17
DKK4
FZD5
FZD7
PITX2
FZD8
WNT3
AXIN2
APC
FZD4
CDH1
SOX2
CD24
NANOG
LGR4
ID2
FZD3
TCF4
TERT
JAG1
DKK1
LGR5
WNT4
WNT11
RSPO4
LEF1
WNT5B
CD44
FGF18
SOX9
b
HIDEN-KO log2(TPM+1)
WT log2(TPM+1)
MYC
DKK4
ID2
CDH1
SOX2
LBH
SOX17
FZD5
PORCN
FZD8
PITX2
TERT
WNT11
JAG1
WNT8A
FZD4
RSPO4
APC
WNT2B
DKK1
DKK2
LGR5
RSPO3
c
TPM
ES
DE
FZD5
LGR5
d
TPM
WT
HIDEN-KO
n.s.
FZD5
LGR5
e
HIDEN-pulldown
% input
Exp1
Exp2
Exp3
MALAT1
GAPDH
FZD5
IMP1
f
Relative expression
WT
IMP1-KO#1
IMP1-KO#2
IMP1-KO#3
FZD5
g
FZD5
Exon1
ATG
Exon2
PAM
sgRNA1
sgRNA2
PAM
GACCCATCCGCGCCGCCCTCGCTGTTGC
CTGGGTAGGCGCGGCGGGAGCGACAACG
398bp
TGCATGGATTACAACCGCAGCGAGGCC
ACGTACCTAATGTTGGCGTCGCTCCGG
WT:
Allele1: ATTCCT // ATG // CCATCCGCGCCGCCCTCGCTGTT // CATGGATTACAACCGCAAGCGAGGCC // GCCAAG
Allele2: ATTCCT // ATG // CCATCCGCGCCGCCCTCGCTGTT // CATGGATTACAACCGCAAGCGAGGCC // GCCAAG
FZD5-KO:
KO#1: -330/-330
Allele1: ATTCCT..........................del/296bp..............................// CATGGATTACAACCGCA.....del/34bp......// GCCAAG
Allele2: ATTCCT..........................del/296bp..............................// CATGGATTACAACCGCA.....del/34bp......// GCCAAG
KO#2: -143/-143
Allele1: ATTCCT // ATG // CCATC.........del/12bp.........GCTGTT // ....del/131bp...........AGCGAGGCC // GCCAAG
Allele2: ATTCCT // ATG // CCATC.........del/12bp.........GCTGTT // ....del/131bp...........AGCGAGGCC // GCCAAG
h
Relative expression in DE
WT
FZD5-KO#1
FZD5-KO#2
FZD5
i
shC
FZD5-sh1
FZD5-sh2
FZD5-sh3
FZD5-sh4
NANOG SOX2 DAPI
SSEA4 OCT4 DAPI
j
Knockdown efficiency
shC
FZD5-sh1
FZD5-sh2
FZD5-sh3
FZD5-sh4
FZD5
k
shC
FZD5-sh1
FZD5-sh2
FZD5-sh3
FZD5-sh4
FOXA2 SOX2 DAPI
SOX17 NANOG DAPI
l
% CXCR4+ by cell cytometry
shC
FZD5-sh1
FZD5-sh2
FZD5-sh3
FZD5-sh4
m
Endoderm
Mesoderm
Pluripotency
Relative expression in DE
shC
FZD5-sh1
FZD5-sh2
FZD5-sh3
FZD5-sh4
SOX17
FOXA2
CXCR4
MIXL1
T
SOX2
OCT4
NANOG
n.s.

Figure S7. *HIDEN* promotes WNT pathway through FZD5.

(**a**) The plot of WNT-associated gene expression during DE differentiation from human ESCs.

(**b**) The plot of WNT-associated gene expression upon *HIDEN*-KO in DE cells.

(**c-d**) The TPM value of *FZD5*, *LGR5* during DE differentiation (**c**), and in *HIDEN*-KO DE cells compared to wildtype (**d**) (n=3).

(**e**) The enrichment of mRNAs or lncRNAs pulled down by *HIDEN* in HUES8 DE cells (normalized by input). Three independent experiments were shown.

(**f**) The relative expression of *FZD5* in wildtype or IMP1 knockout DE cells, as shown by RT-qPCR (n=3).

(**g**) The genotype of FZD5-knockout ESC lines. The sgRNA targeting sequences, PAM sequence and deleted nucleotides were indicated.

(**h**) The knockout efficiency of FZD5 in wildtype or FZD5-knockout DE cells, as shown by RT-qPCR (n=3).

(**i**) Immunofluorescent staining of pluripotency gene markers (OCT4, SSEA4, NANOG, SOX2) in shRNA control or FZD5 knockdown ESCs. Scale bar=50 μm.

(**j**) The determination of knockdown efficiency of FZD5 (n=3).

(**k**) Immunofluorescent staining of DE markers (SOX17, FOXA2) and pluripotency markers (NANOG, SOX2) in shC and FZD5-KD DE cells. Scale bar=50 μm.

(**l**) Flow cytometry analysis of $CXCR4^{+}$ cells in shC or FZD5-KD DE cells (n=3).

(**m**) RT-qPCR analysis of RNA levels of representative endoderm genes, mesoderm genes and pluripotency genes in wildtype or FZD5 knockdown DE cells (n=3).
