# Supplementary material for: A desert lncRNA HIDEN regulates human endoderm differentiation via interacting with IMP1 and stabilizing FZD5 mRNA
Source: Genome Biol. 2023 Apr 24;24:92. doi: 10.1186/s13059-023-02925-w (PMC10124006; doi:10.1186/s13059-023-02925-w)
Supplement: Supplementary file 3 — HIDEN sequence. [file 13059_2023_2925_MOESM3_ESM.pdf]

HIDEN sequence 1 (1045nt):

GCTGACTCTCTTTTAGGACTCAGCCCACCTGCACCCAGGTGATTAAAAAG

CTTTATTGCTCACACAAAGCCTGTTGGTGGTCTCTTTACATGGATGCACAA

GACACTCTTGACTTTGATTTTCTGCAGTATGAATATGGGACATGCATGAAA

TTTGGTGCCGTGACTCAGATCGGGGGACCTCCTTTGGGAGATCAATCCCCT

GTCCTCCTGATCTTTGCTCCATGAGAAAGATCCACCTACAACCTCAAGTCC

TCAGACTGACCAGCCCAAGAAACATCTCACCAATTTCAAATCCGGACCCT

ACTGAAAATCAGACTGTTCAACTCACCTGGCAGCCACTCCCAGAGCCCCT

GGAACTCTGGCCCAAGGCTCTCTGACTGACTCCTTCCCAGATCTTCTCAGC

TTAGCAGCTGAAGACTGACACTGCCCAATCGCCTCAGAAGCCTACAGGAC

CATCACAGATGCTCTAGGTAACTCTCACAGTGGAAGAAAAGTAATGGAAT

GCTACAGCCTAGTCAGTTTTGAGAAAAGGCTAACCAACAACAACAACAAC

AAATCAcAGTCATGCTCAGATTGCACATGAACAAAAAAATGTTGGAGAGA

AACCATTGAAATAACTCACCGGTCCTCTTGTGTTGCTGCTTATCTGATTAT

CTGAGGTCAAGGGAACTGAGGACAACAAAGGAAATTAGGTCTTTCATAGT

TCTCATTAAATGCTGGAGGAGTCTGACTACTGCTGAGCTGAGCACTTCAG

CGGCTTTGGAAAAGGAAGTGTGGTTACTTATTTCACCACCTTCATTGTGCT

GGAGGAAATAAGAAATAGCTCTGAGACTAATAATAACAGAAACGGCATC

CACAGCAGCAGAGCCCAGTTGCGCTTCCTAAAGGCAGTATAAATAATTAA

GTGTCTCTATAGGCAAGCCACTAGGAATAAACGAAAAAGAGCTGCAATTG

CTGATGTGGTTTCACCCCAGTCCTGCCCATGTTTCTTAACAGGAAAGGGAG

TTGCAATaCCAATAATAAAATCTTAGAGCTGGAAAGAA

HIDEN sequence 2 (1169nt):

GCTGACTCTCTTTTAGGACTCAGCCCACCTGCACCCAGGTGATTAAAAAG

CTTTATTGCTCACACAAAGCCTGTTGGTGGTCTCTTTACATGGATGCACAA

GACACTCTTGACTTTGATTTTCTGCAGTATGAATATGGGACATGCATGAAA

TTTGGTGCCGTGACTCAGATCGGGGGACCTCCTTTGGGAGATCAATCCCCT

GTCCTCCTGATCTTTACTCCATGAGAAAGATCCACCTACAACCTCAAGTCC

TCAGACTGACCAGCCCAAGAAACATCTCACCAATTTCAAATCCGGACCCT
ACTGAAAATCAGACTGTTCAACTCACCTGGCAGCCACTCCCAGAGCCCCT
GGAACTCTGGCCCAAGGCTCTCTGACTGACTCCTTCCCAGATCTTCTCAGC
TTAGCAGCTGAAGACTGACACTGCCCAATCGCCTCAGAAGCCTACAGGAC
CATCACAGATGCTCTAGGTAACTCTCACAGTGGAAGACTCAGCCCGCCTG
CACCCAGGTGAAATAAACAGCCATGTTGCTCACACAAAGCCTGTTTGGTG
GTCTCTTCACACGGACGTGCATGAAACTACCTACACCCATACGGAAGGGA
ACAAATCATGAAAAGTAATGGAATGCTACAGCCTAGTCAGTTTTGAGAAA
AGGCTAACCAACAACAACAACAACAAATCACAGTCATGCTCAGATTGCAC
ATGAACAAAAAAATGTTGGAGAGAAACCATTGAAATAACTCACCGGTCCT
CTTGTGTTGCTGCTTATCTGATTATCTGAGGTCAAGGGAACTGAGGACAAC
AAAGGAAATTAGGTCTTTCATAGTTCTCATTAAATGCTGGAGGAGTCTGA
CTACTGCTGAGCTGAGCACTTCAGCGGCTTTGGAAAAGGAAGTGTGGTTA
CTTATTTCACCACCTTCATTGTGCTGGAGGAAATAAGAAATAGCTCTGAG
ACTAATAATAACAGAAACGGCATCCACAGCAGCAGAGCCCAGTTGCGCTT
CCTAAAGGCAGTATAAATAATTAAGTGTCTCTATAGGCAAGCCACTAGGA
ATAAACGAAAAAGAGCTGCAATTGCTGATGTGGCTTCACCCCAGTCCTGC
CCATGTTTCTTAACAGGAAAGGGAGTTGCAATGCCAATAATAAAATCTTA
GAGCTGGAAAGAA
