# Supplementary material for: A desert lncRNA HIDEN regulates human endoderm differentiation via interacting with IMP1 and stabilizing FZD5 mRNA
Source: Genome Biol. 2023 Apr 24;24:92. doi: 10.1186/s13059-023-02925-w (PMC10124006; doi:10.1186/s13059-023-02925-w)

Shown in Figure 2a

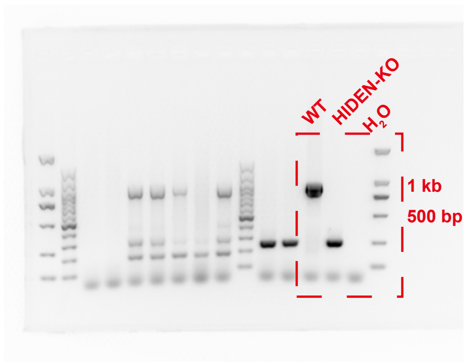

Shown in Figure S2a

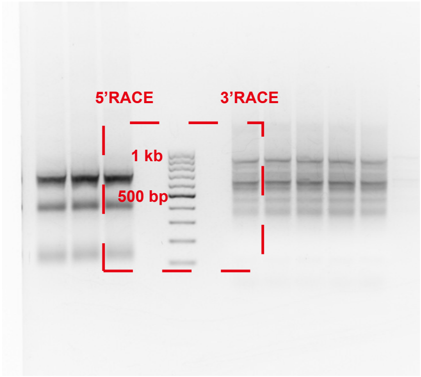

Shown in Figure 3g,S4d

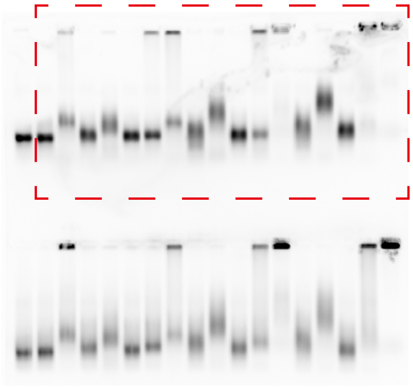

Shown in Figure 2d

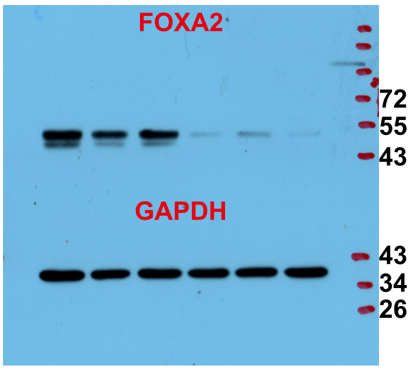

Shown in Figure 2d

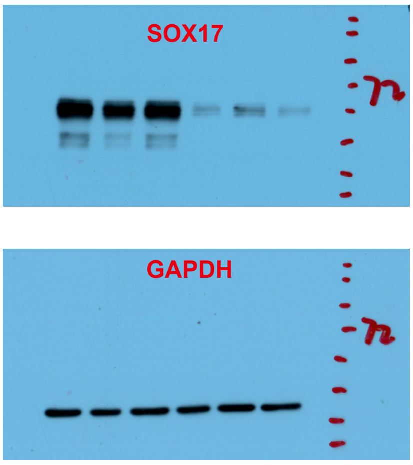

Shown in Figure 3c

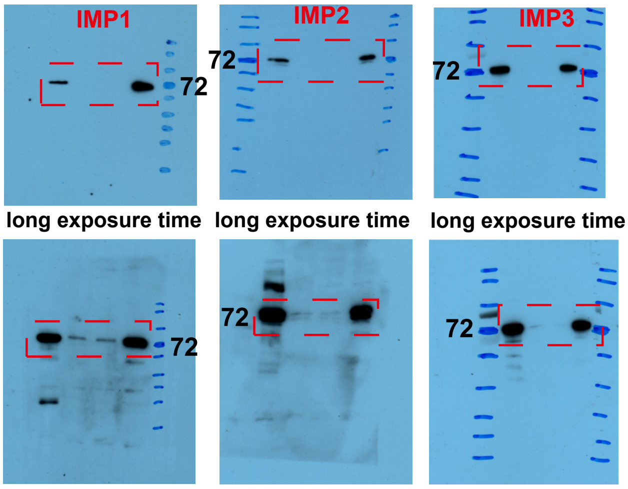

Shown in Figure 3e

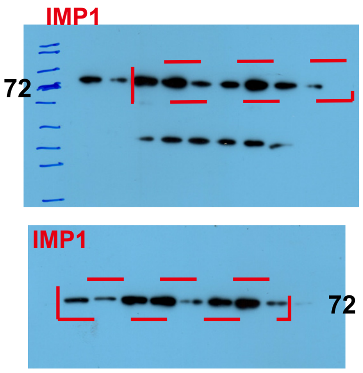

Shown in Figure S4a

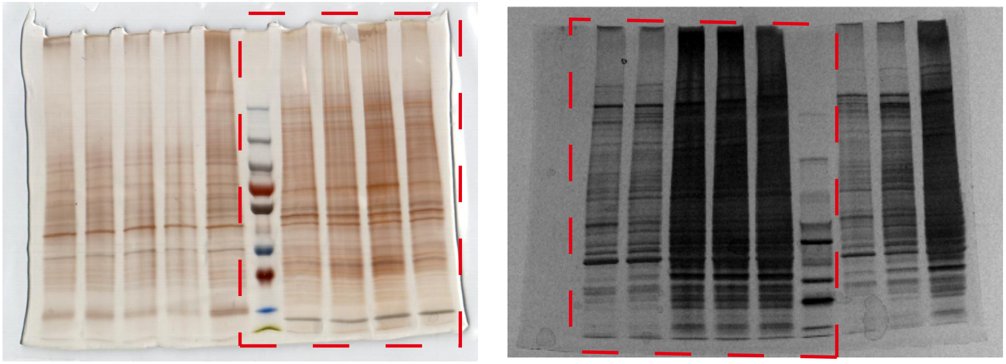

Shown in Figure S4c

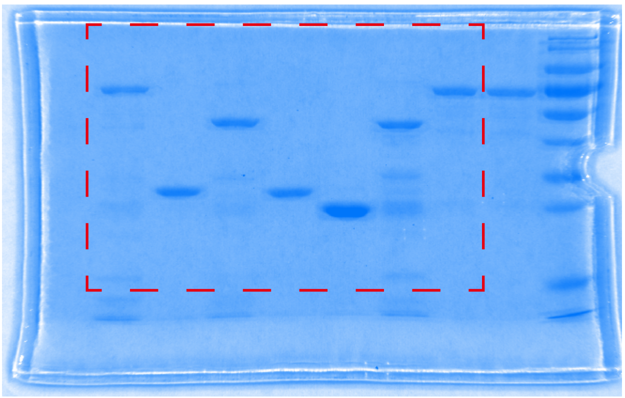

Shown in Figure 5c

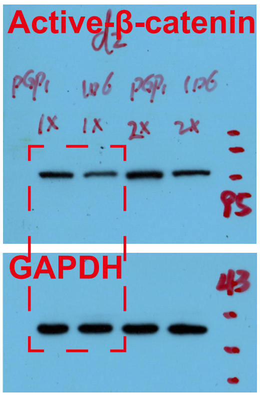

Shown in Figure

5c S6e

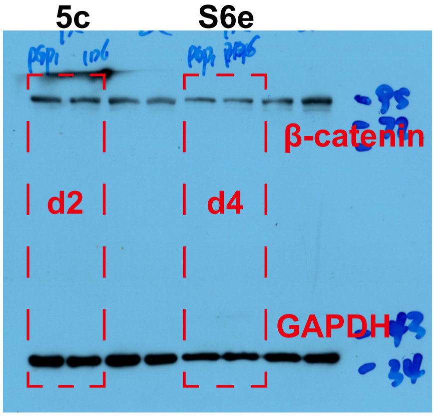

Shown in Figure S5c

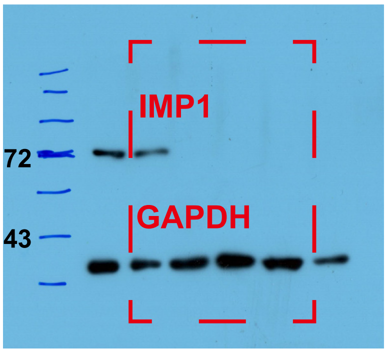

Shown in Figure S6a

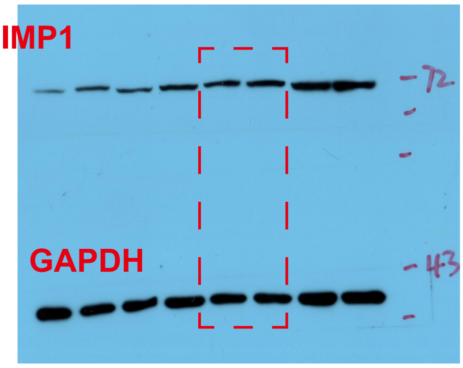

Shown in Figure S6b

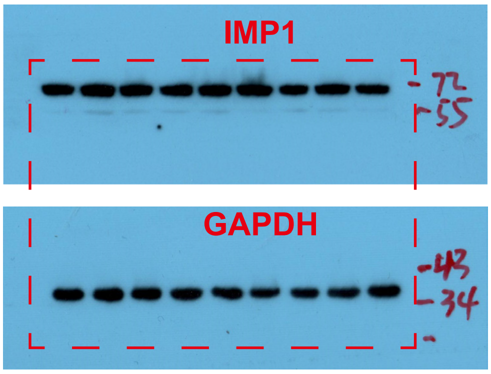

Shown in Figure 5e

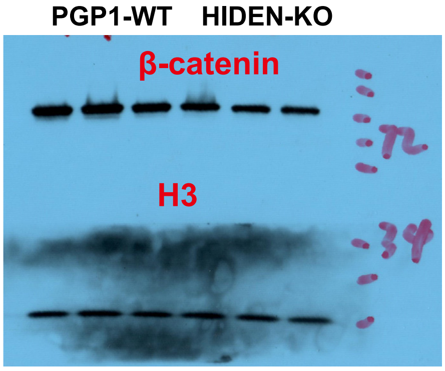

Shown in Figure S6e

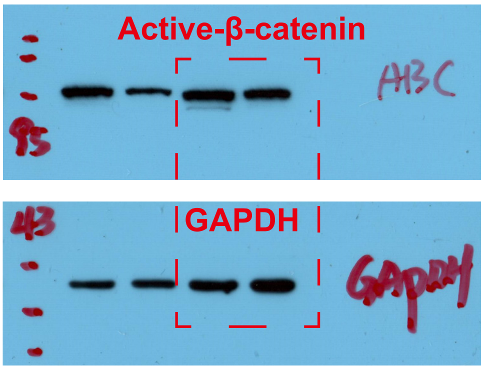

Shown in Figure S6f

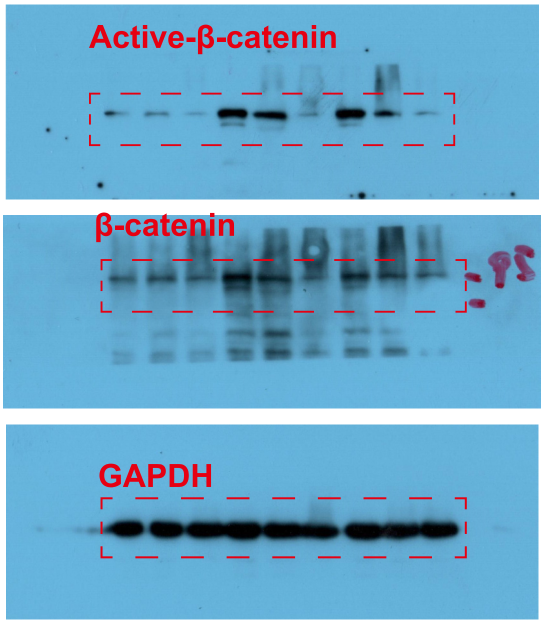

Supplement: Supplementary file 11 — Uncropped images for western blots in this manuscript. [file 13059_2023_2925_MOESM11_ESM.pdf]
