# Supplementary material for: A desert lncRNA HIDEN regulates human endoderm differentiation via interacting with IMP1 and stabilizing FZD5 mRNA
Source: Genome Biol. 2023 Apr 24;24:92. doi: 10.1186/s13059-023-02925-w (PMC10124006; doi:10.1186/s13059-023-02925-w)
Supplement: Supplementary file 12 — Review history. [file 13059_2023_2925_MOESM12_ESM.docx]

**1^st^ round**

**Reviewer 1**

The lncRNA HIDEN was a newly characterized lncRNA during human PSC differentiation whose transcriptional position is more than 50 kb away from the protein coding region, classified as "desert lncRNAs". The authors utilized RNA pull-down assay to identify IGF2BP1 (IMP1) as the key RNA binding protein that interacting with HIDEN and controls the endoderm differentiation by stabilizing FZD5 mRNA to promote WNT activity. The critical role of HIDEN/IMP1/FZD5 axis in human early endoderm differentiation was demonstrated. I have the following comments:

Major comments:
1. HIDEN-KD or -KO inhibited PSC differentiating to DE cells accompanied by the persistent high expression of stemness genes including OCT4, SOX2 and NANOG, as shown in Figure 2C and Figure S2 d-g. However, the continuous high expression of stemness genes is an important factor that hinders the differentiation of PSCs into DE cells. Whether the maintenance of stemness genes was caused by HIDEN deletion? How does HIDEN maintain the high expression of pluripotent genes during DE differentiation?
2. In Figure 6d-e, the authors demonstrated that "the disruption of HIDEN could significantly diminish the interaction of IMP1 protein and FZD5 mRNA" and "the expression of FZD5 was much lower upon HIDEN knockout". The authors should confirm whether the IMP1-RIP enrichment of FZD5 mRNA was due to the downregulated FZD5 mRNA expression.
3. The KD or KO of both IMP1 and HIDEN can induce FZD5 mRNA downregulation. And the author also found the interaction of IMP1 with HIDEN and FZD5. However, these evidence cannot conclude the HIDEN/IMP1/FZD5 axis. To make the conclusion more solid, IMP1 overexpression as rescue experiments in HIDEN-KO cells should be included.
4. The structure of HIDEN was predicted as Figure S3, and the stem-loop structures within 251-650 nt fragment was found to be responsible for the binding with IMP1. However, whether the antisense sequence of HIDEN have the same stem-loop structures as HIDEN? The antisense sequence was used as negative control in RNA pulldown assay in Figure 3e and Figure s3a. In Figure 3e, the antisense sequence showed obvious enrichment of IMP1. Therefore, the antisense sequence of a lncRNA may not be an appropriate negative control for they may have the same stem-loop structures. Mutations that can destroy the key stem-loop structurers of HIDEN may be more suitable for negative control.
5. Why the antisense RNA-pulldown bands in Figure 3c and 3e are different?
6. As a m6A reader protein, IGF2BP1 regulates many mRNAs in PSCs. As HIDEN regulates IGF2BP1 to stabilizing FZD5 during DE differentiation, does HIDEN/IGF2BP1 axis have effects on other mRNAs like OCT4, SOX2 and NANOG in DE cells?

Minor comments:
1. Many figures are lacking group legend, e.g. Figure 5g, h, j.
2. The naming of HIDEN ("human IMP1-associated "desert" definitive endoderm lncRNA") should be written in the manuscript.

**Reviewer 2**

Lu et al report on a intergenic lncRNA, called HIDEN, as a novel player in human edoderm differentiation. They demonstrate a role for HIDEN in regulating differentiation potential and identified IMP1 as the interaction partner through which HIDEN regulates expression of the WNT pathway. The results are properly presented and experiments are conducted using the necessary controls. The authors apply orthogonal methods throughout the results section to validate their observations, which results in convincing conclusions.

Main comments:

Is HIDEN efficiently spliced and/or conserved? It would be interesting to include a RNA-seq coverage plot of HIDEN and indicate sequence conservation levels of the HIDEN exons.

Figure 4f: Is the overlap between DE genes observed in HIDEN KO and IMP1 KO cells significant?

Line 297: Authors refer to 'peaks'? Not clear what is meant here?

Figure 6e: What is the impact of HIDEN KO on IMP1 binding to other RNAs? Is this effect (ie reduced binding of IMP1 to target RNA) also observed for other RNAs, or only for FZD5?

Does HIDEN upregulation precede FZD5 upregulation in the differentiation series presented in Figure 1f?

To better understand the role of HIDEN in the IMP1/HIDEN/FZD5 complex, it would be interesting to investigate if HIDEN also binds FZD5 mRNA.

Minor comment:

Line 117: Authors mention 'differentially expressed' lncRNAs. I assume they mean 'expressed' lncRNAs?

**Authors’ response**

Experiment-wise, we performed embryoid body experiments to further confirm the importance of HIDEN for endoderm differentiation, showing decreased endoderm differentiation in HIDEN-KO cells compared with wildtype under spontaneous differentiation condition (Figure S2f); we provided immunofluorescent images of day- by-day dynamic endoderm differentiation, showing the delayed differentiation process (Figure S3e-f); we performed cell proliferation analysis in wildtype and HIDEN-KO PSCs, showing little influence on cell proliferation upon HIDEN-KO (Figure S2e); we obtained the pulldown data to support the interaction between HIDEN and FZD5 (Figure S7e);

Analysis-wise, we accordingly performed the overlap and correlation analysis of transcriptome indicating there are a significant overlap between HIDEN-affected genes and IMP1-affected genes (Figure 4f-g); we provided the conservation analysis of HIDEN, indicating it is likely a primate-specific lncRNA; more importantly, we identified another isoform with 1169 nucleotides and sharing the same promoter as the previous one (Figure S2b).

We hope that the reviewers could satisfy with our revision. Our point-by-point responses to reviewers’ concern are listed below:

Reviewer #1: The lncRNA HIDEN was a newly characterized lncRNA during human PSC differentiation whose transcriptional position is more than 50 kb away from the protein coding region, classified as "desert lncRNAs". The authors utilized RNA pull-down assay to identify IGF2BP1 (IMP1) as the key RNA binding protein that interacting with HIDEN and controls the endoderm differentiation by stabilizing FZD5 mRNA to promote WNT activity. The critical role of HIDEN/IMP1/FZD5 axis in human early endoderm differentiation was demonstrated. I have the following comments:

Major comments:

1. HIDEN-KD or -KO inhibited PSC differentiating to DE cells accompanied by the persistent high expression of stemness genes including OCT4, SOX2 and NANOG, as shown in Figure 2C and Figure S2 d-g. However, the continuous high expression of stemness genes is an important factor that hinders the differentiation of PSCs into DE cells. Whether the maintenance of stemness genes was caused by HIDEN deletion? How does HIDEN maintain the high expression of pluripotent genes during DE differentiation?

Response: Regarding the relatively high expression of stemness genes in differentiated HIDEN-KD or -KO cells, we think it is mainly due to the low differentiation efficiency and/or delayed differentiation process, as a phenotype of impaired lineage differentiation. The expression levels of stemness genes in differentiated HIDEN-KD or -KO cells were indeed higher than differentiated control or wildtype cells, but still significantly lower than undifferentiated ESCs as shown below. Similar circumstances (relatively high expression of stemness genes in HIDEN- KD or -KO differentiated cells) were observed in other reported endoderm-functional lncRNAs, such as DEANR1 (Cell Rep. 2015;11(1):137-148), GATA6-AS1 (Stem Cell Reports 2020;15(3):694-705), and T-REX17 (Elife 2023;12:e83077). In addition, we monitored the expression of DE marker genes (SOX17, FOXA2) day-by-day during the four days of DE differentiation (scale bar=100 μm). The percentages of SOX17- or FOXA2-positive DE cells in HIDEN-KD or -KO cells are indeed always less than control wildtype cells, but continue to grow during differentiation, which indicated delayed DE differentiation. The delayed differentiation process usually accompanies with slower downregulated expression of pluripotent genes. We added this part in Figure S3e-f.

Regarding the relationship between HIDEN and pluripotent genes, we first observed the relatively low expression of HIDEN in PSCs (Figure 1g), indicating that HIDEN might not be required for the maintenance of stemness genes, partially supported by the observation that HIDEN-KO or -KD PSCs are normal based on pluripotency gene expression and cell proliferation (Figure S2d-e, S3a-c). In addition, based on our working model, HIDEN plays a post-transcriptional role (in regulating mRNA stability by interacting with IMP1), while abundant studies have revealed that the downregulation of stemness genes during differentiation and pluripotency exit is remarkably regulated at the transcription level by multiple chromatin modifiers or transcription factors (Nucleic Acids Res. 2018;46(13):6592-6607; Nucleic Acids Res. 2019;47(21):11114-11131; Nature 2018;555(7695):256-259). Collectively, we suspected that HIDEN might play a weak role, if not, in regulating stemness genes.

2. In Figure 6d-e, the authors demonstrated that "the disruption of HIDEN could significantly diminish the interaction of IMP1 protein and FZD5 mRNA" and "the expression of FZD5 was much lower upon HIDEN knockout". The authors should confirm whether the IMP1-RIP enrichment of FZD5 mRNA was due to the downregulated FZD5 mRNA expression.

Response: In Figure 6d, to calculate the enrichment of RNA in IMP1-RIP, we first normalized each IP fraction’s Ct average to the input fraction Ct average for ΔCt: (ΔCt[normalized RIP] = (Average Ct[RIP] - (Average Ct[Input] - log2(Input Dilution Factor)), where Input Dilution Factor = (fraction of the input RNA saved), and then calculated the % input for each RIP fraction: % Input = 2^ (−ΔCt[normalized RIP]). Thus, we normalized IP fraction’s Ct to the Input fraction Ct and calculated the percentage of input for RIP fraction (reference as Methods Mol Biol. 2016;1480:73-86), which has already taken account of the input difference.

In IMP1-RIP-seq data, the expression of FZD5 was indeed decreased upon HIDEN knockout. However, we evaluated whether RNA was enriched by IMP1 based on the corresponding input sample using MACS2 software, which uses a dynamic Poisson distribution to effectively capture local biases in the genome, allowing for more robust predictions (Nature 2018;555(7695):256-259; Methods Mol Biol. 2015;1269:293-303; Genome Biol. 2008;9(9):R137). Therefore, the lower expression of different inputs was considered. The following is an example: CD44 was a well-known IMP target gene (EMBO J. 2006;25(7):1456-1468) and its expression was also downregulated in the knockout sample, but it was still identified as an IMP1-bound gene in our dataset.

3. The KD or KO of both IMP1 and HIDEN can induce FZD5 mRNA downregulation. And the author also found the interaction of IMP1 with HIDEN and FZD5. However, these evidence cannot conclude the HIDEN/IMP1/FZD5 axis. To make the conclusion more solid, IMP1 overexpression as rescue experiments in HIDEN-KO cells should be included.

Response: We thank the reviewer for this suggestion. We accordingly conducted the IMP1 overexpression experiment using the lentiviral system to achieve higher transfection efficiency. We successfully overexpressed IMP1, but we found IMP1 overexpression caused blocked DE differentiation (shown below), similar to the reports that over-activation of WNT signaling (the downstream of IMP-HIDEN) is inhibitory for DE differentiation (Figure 5g) (Cell Res. 2013;23(1):122-30; Stem Cell Rev Rep. 2014;10(4):480-493; In Vitro Cell Dev Biol Anim. 2015;51(1):1-8). That may indicate: 1) the IMP1 dosage, just like WNT activity, may be critical for efficient DE differentiation; 2) the impact of excessive IMP1 in pluripotent state could affect the following lineage differentiation since IMP1 is already highly expressed in PSCs, unlike HIDEN. We have another ongoing project aiming to reveal the different roles of individual IMP protein in stem cells and different lineages (three IMPs are highly expressed in PSCs but exhibit distinct patterns during three germ layer differentiation).

In fact, when we utilized the WNT agonist to rescue the phenotype caused by IMP1-knockout or HIDEN-knockout, low concentration (0.5 μM) worked quite well, while the high concentration of CHIR-99021 (2 μM or more) inhibited DE differentiation in wildtype (Figure 5g). Therefore, we believe the HIDEN-knockout rescued by IMP1 would require precise control of IMP1 expression, such as doxycycline-inducible expression of IMP1 which takes a much longer time. In addition, HIDEN interacted with IMP1 protein and together regulated FZD5 mRNA stability in our working model. Without HIDEN, the binding of IMP1 protein on FZD5 RNA was dramatically decreased (Figure 6d-e). Moreover, the FZD5 mRNA became unstable upon knocking out HIDEN (Figure 6f). It’s further supported by the examination of WNT activity by 7xTCF reporter or beta-catenin expression analysis (Figure 5f, Figure S6i), since FZD5 is an important receptor of WNT signaling pathway. Therefore, activation of the final target WNT signal, by either overexpressing FZD5 or adding proper WNT agonist, was expected to be able to rescue the defected endoderm differentiation caused by HIDEN or IMP1 knockout. We utilized the WNT agonist CHIR to establish this functional link (Figure 5g-j, Figure S6h).

4. The structure of HIDEN was predicted as Figure S3, and the stem-loop structures within 251-650 nt fragment was found to be responsible for the binding with IMP1. However, whether the antisense sequence of HIDEN have the same stem-loop structures as HIDEN? The antisense sequence was used as negative control in RNA pulldown assay in Figure 3e and Figure s3a. In Figure 3e, the antisense sequence showed obvious enrichment of IMP1. Therefore, the antisense sequence of a lncRNA may not be an appropriate negative control for they may have the same stem-loop structures. Mutations that can destroy the key stem-loop structurers of HIDEN may be more suitable for negative control.

Response: We thank the reviewer for raising the issue of the structure of HIDEN and antisense control. Since the RNA structure is quite difficult to resolve experimentally (Nat Methods 2022;19(10):1193-1207; Signal Transduct Target Ther. 2022;7(1):58), we used RNAfold and Mfold to calculate the secondary structures of HIDEN and the antisense, shown as below. We found the stem-loop structures within 250-650 nt were obviously different from each other, especially for the 450-650 nt position. This prediction is consistent with our experimental data which showed 250- 650 nt fragment is responsible to the interaction between HIDEN and IMP1 (Figure 3e). Regarding the control issue, for the most important experiment we also included luciferase transcript and another unrelated lncRNA (GATA6-AS1) as controls in the silver staining of HIDEN pulldown (followed by Mass-Spec) (Figure S4a), and found the enriched band around 72 kD pulled-down by HIDEN was the most obvious. In addition, the relative higher enrichment of IMP1 pulled down by HIDEN compared to antisense or beads group was confirmed by multiple assays (Exp1 was shown in Figure 3c).

5. Why the antisense RNA-pulldown bands in Figure 3c and 3e are different?

Response: We are sorry for the unclear statement. We conducted separate RNA- pulldown experiments, and the cell lysates were collected from differentiated human DE cells (Figure 3c) and 293T cells (Figure 3e) respectively. The difference in cell lysate, exposure time or other experimental factors may contribute to the difference in the background signal (antisense sample). The images in Figure 3c/3e with long or short exposure time were shown below, indicating the exposure time could indeed result in certain background signal. Nevertheless, the much stronger enrichment of IMP1 by 250-650 nt fragment of HIDEN was repeatedly observed.

6. As a m6A reader protein, IGF2BP1 regulates many mRNAs in PSCs. As HIDEN regulates IGF2BP1 to stabilizing FZD5 during DE differentiation, does HIDEN/IGF2BP1 axis have effects on other mRNAs like OCT4, SOX2 and NANOG in DE cells?

Response: We thank the reviewer for raising this issue. As the reviewer mentioned, HIDEN/IGF2BP1 axis indeed regulated many mRNAs in DE cells, which was similar to that of IGF2BP1 in PSCs (Cell Rep 2016;15:666-679). In Figure 5a, we identified 1207 genes bound by IMP1 (determined by IMP1-RIP-seq) and affected by HIDEN (determined by RNA-seq of HIDEN-knockout and wildtype samples) (also shown below). Furthermore, by comparing the binding on these genes by IMP1 RIP-seq in HIDEN-knockout and wildtype cells, we found 749 genes, including FZD5, exhibited the reduced enrichment of IMP1 upon HIDEN knockout. Among these genes, we observed significantly enriched WNT signaling pathway-associated genes (shown below), which supported WNT signaling pathway as the downstream of HIDEN/IMP1. Next, we checked the 749 genes and pluripotent genes (including OCT4, SOX2 and NANOG) were not in the list. In fact, IMP1 did not even bind to these mRNAs. The result was consistent with unaltered pluripotency gene expression upon IMP1-KO in human PSCs (Figure S5d). This data suggested HIDEN knockout during endoderm differentiation affected a serial of genes besides of FZD5, but not pluripotent genes.

We further investigated these 749 genes, and the GO analysis (shown above) showed these genes enriched in WNT signaling which was an important and well-known regulator for endoderm differentiation. Combined with the rescue result using WNT agonist, we next focused on the WNT genes and identified FZD5 as the major functional target mediating the impaired endoderm differentiation phenotype in HIDEN-knockout PSCs (Figure 6).

Minor comments:

1. Many figures are lacking group legend, e.g. Figure 5g, h, j.

Response: We are sorry for the inconvenience. We have checked and added the group legend in every figure.

2. The naming of HIDEN ("human IMP1-associated "desert" definitive endoderm lncRNA") should be written in the manuscript.

Response: We have added the naming of HIDEN in the manuscript.

Reviewer #2: Lu et al report on a intergenic lncRNA, called HIDEN, as a novel player in human endoderm differentiation. They demonstrate a role for HIDEN in regulating differentiation potential and identified IMP1 as the interaction partner through which HIDEN regulates expression of the WNT pathway. The results are properly presented and experiments are conducted using the necessary controls. The authors apply orthogonal methods throughout the results section to validate their observations, which results in convincing conclusions.

Main comments:

Is HIDEN efficiently spliced and/or conserved? It would be interesting to include a RNA-seq coverage plot of HIDEN and indicate sequence conservation levels of the HIDEN exons.

Response: We thank the reviewer for raising this issue. Regarding species conservation, we have checked the Multiz Alignments of 100 Vertebrates from UCSC Genome Browser on Human (hg38) and the basewise conservation (phyloP) values were shown below. The genomic location of HIDEN is not that conserved, but the last exon showed a certain conservation tendency only in mammals.

Regarding the splicing, we accordingly presented the RNA-seq coverage plot of HIDEN from the three PSC lines used in this study. Very interestingly, HUES8 showed nice coverage with our RACE'd sequence, but there seems to be an extra exon shown in the other two lines (H9 and PGP1). The extra exon (124-nt) was indeed detected in differentiated H9 and PGP1 endoderm cells but not in HUES8 by RT-PCR.

Therefore, we re-checked our RACE assay. The gene specific primer 1 (GSP1, located in the last exon) and GSP2 (located in second exon) were designed based on the common region of all annotated HIDEN isoforms and then used to obtain the 5’ end and 3’ end of HIDEN transcript using HUES8 endoderm cells. The serial experiments resulted in the identification of the 1045-nt transcript with four exons. We then confirmed the existence of the 1045-nt full-length of HIDEN by directed PCR with cDNA from all the three cell lines (validated by Sanger sequencing). Interestingly, we did detect the isoform containing all the five exons in PGP1 cells and sequenced the long isoform (1169-nt). We have accordingly updated the sequences of HIDEN (revised Supplementary Material 1). Nevertheless, HIDEN-KO showed efficient knockout of all exons (as shown below), and the targeting of shRNA or qPCR primers both included the isoform consideration (Figure S2b, Figure 2a), which demonstrated the effective disruption of HIDEN transcripts.

Figure 4f: Is the overlap between DE genes observed in HIDEN KO and IMP1 KO cells significant?

Response: We thank the reviewer for the nice suggestion. We calculated the correlation of definitive endoderm (DE) genes (the list contains 591 DE signature genes and is generated from our previous publication: Stem Cell Reports 2020;15(3):694-705) between HIDEN-KO and IMP1-KO. The results showed that DE genes were significantly correlated between HIDEN-KO and IMP1-KO. Then, we extracted the differentially expressed DE genes from HIDEN-KO and IMP1-KO RNA-seq data. The results showed that 131 genes were significantly overlapped between HIDEN-KO and IMP1-KO differentially expressed DE genes (p=1.33e-10). The heatmap showed that the most overlap DE genes were downregulated in HIDEN-KO and IMP1-KO cells. In addition, we also compared the differentially expressed genes due to HIDEN knockout or IMP1 knockout. HIDEN-affected genes were also significantly correlated with IMP1-affected genes in endoderm cells (Figure 4f, also shown below). Moreover, most of these co-regulated genes exhibited a similar change trend upon knocking out HIDEN or IMP1 (Figure 4g, also shown below). These results further indicated that HIDEN and IMP1 regulated the similar set of genes.

Line 297: Authors refer to 'peaks'? Not clear what is meant here?

Response: We are sorry for the unclear statement. For IMP1 RIP-seq data, we first calculated the enrichment of IMP1-bound peaks by MACS2 software (similar to ChIP-seq data processing, which uses a dynamic Poisson distribution to effectively capture local biases in the genome, allowing for more robust predictions: Nature 2018;555(7695):256-259; Methods Mol Biol. 2015;1269:293-303), and then annotated significantly enriched peaks to corresponding genes. We had corrected the writing to make the description more accurate. Meanwhile, we also added the figure of peaks and annotated genes in our revised version (Figure S6c-d).

Figure 6e: What is the impact of HIDEN KO on IMP1 binding to other RNAs? Is this effect (ie reduced binding of IMP1 to target RNA) also observed for other RNAs, or only for FZD5?

Response: We identified 1207 genes bound by IMP1 (determined by IMP1-RIP- seq) and affected by HIDEN (determined by RNA-seq of HIDEN-knockout and wildtype samples) (revised Figure 5a). To address the Reviewer's question, we further analyzed the binding on these genes by IMP1 RIP-seq in HIDEN-knockout and wildtype cells. Among these 1207 genes, 749 genes, including FZD5, exhibited the reduced enrichment of IMP1 upon HIDEN knockout. This data suggested HIDEN knockout during endoderm differentiation affected a serial of genes besides of FZD5. We further investigated these 749 genes and the GO analysis showed these genes enriched in WNT signaling which was an important and well-known regulator for endoderm differentiation. Combined with the rescue result using WNT agonist, we next focused on the WNT associated genes and identified FZD5 as the major target by mRNA stability examination and phenocopying experiments (Figure 6). FZD5 is not the only gene regulated by HIDEN/IMP1 stated as above, but our data indicated it was a functional target mediating the impaired endoderm differentiation phenotype in HIDEN-knockout PSCs.

Does HIDEN upregulation precede FZD5 upregulation in the differentiation series presented in Figure 1f?

Response: We accordingly examined the expression dynamics of FZD5 and HIDEN during endoderm differentiation (shown below). Of note, the expression level of HIDEN was much higher than FZD5 (the TPM of HIDEN and FZD5 is 2937±171 and 44.8±16.2, respectively) based on our RNA-seq data in differentiated endoderm cells of PGP1 cell line. In addition, we checked the expression of HIDEN and FZD5 in another RNA-seq dataset including different time points during differentiation (GSE168625; FPKM of D0-D2-D5: D0 for PSC, D5 for differentiated definitive endoderm, and D2 for intermediate state), which showed similar result that HIDEN is much higher expressed during DE differentiation.

To better understand the role of HIDEN in the IMP1/HIDEN/FZD5 complex, it would be interesting to investigate if HIDEN also binds FZD5 mRNA.

Response: We gratefully thank the reviewer for this suggestion. Although the measurement of direct interaction between two RNAs is difficult, we have tried some assays during this revision. We first performed the HIDEN RNA pulldown assay followed by RNA extraction. RT-qPCR from three batches of independent HIDEN pulldown shown below indicated certain enrichment of FZD5 mRNA compared to other control RNAs, such as MALAT, GAPDH and IMP1 (percentage of input was calculated).

We next performed the in vitro EMSA assay using Cy5-HIDEN and Cy3-FZD5 transcripts together with IMP1 protein (we failed in observation of shift using only two RNAs). We found the FZD5-UTR1 but not FZD5-UTR2 showed obvious overlap (marked by the white arrow) between Cy5 and Cy3 signal, supporting that IMP1 and HIDEN and FZD5 might form a complex to regulate FZD5 mRNA stability. The data shown above together supported that there might be an interaction between HIDEN and FZD5 transcripts. However, we realized that these data were not sufficient to conclude HIDEN directly interacted with FZD5 RNA. More controls and mutations based on RNA-RNA structure modeling or elucidation, which need much more efforts and collaborations, would eventually address this issue.

Minor comment:

Line 117: Authors mention 'differentially expressed' lncRNAs. I assume they mean 'expressed' lncRNAs?

Response: We are sorry for the mistake and have corrected it accordingly.

Reference

1. Jiang W, Liu Y, Liu R, Zhang K, Zhang Y. The lncRNA DEANR1 facilitates human endoderm differentiation by activating FOXA2 expression. Cell Rep. 2015;11(1):137- 148. doi:10.1016/j.celrep.2015.03.008

2. Yang J, Lu P, Li M, Yan C, Zhang T, Jiang W. GATA6-AS1 Regulates GATA6 Expression to Modulate Human Endoderm Differentiation. Stem Cell Reports. 2020;15(3):694-705. doi:10.1016/j.stemcr.2020.07.014

3. Landshammer A, Bolondi A, Kretzmer H, et al. T-REX17 is a transiently expressed non-coding RNA essential for human endoderm formation. Elife. 2023;12:e83077. Published 2023 Jan 31. doi:10.7554/eLife.83077

4. Kwak S, Kim TW, Kang BH, et al. Zinc finger proteins orchestrate active gene silencing during embryonic stem cell differentiation. Nucleic Acids Res. 2018;46(13):6592-6607. doi:10.1093/nar/gky454

5. Cheng L, Zhang X, Wang Y, et al. Chromatin Assembly Factor 1 (CAF-1) facilitates the establishment of facultative heterochromatin during pluripotency exit. Nucleic Acids Res. 2019;47(21):11114-11131. doi:10.1093/nar/gkz858

6. Bertero A, Brown S, Madrigal P, et al. The SMAD2/3 interactome reveals that TGFβ controls m6A mRNA methylation in pluripotency. Nature. 2018;555(7695):256-259. doi:10.1038/nature25784

7. Gagliardi M, Matarazzo MR. RIP: RNA Immunoprecipitation. Methods Mol Biol. 2016;1480:73-86. doi:10.1007/978-1-4939-6380-5_7

8. Bertero A, Brown S, Madrigal P, et al. The SMAD2/3 interactome reveals that TGFβ controls m6A mRNA methylation in pluripotency. Nature. 2018;555(7695):256-259. doi:10.1038/nature25784

9. Zambelli F, Pavesi G. RIP-Seq data analysis to determine RNA-protein associations. Methods Mol Biol. 2015;1269:293-303. doi:10.1007/978-1-4939-2291-8_18

10. Zhang Y, Liu T, Meyer CA, et al. Model-based analysis of ChIP-Seq (MACS). Genome Biol. 2008;9(9):R137. doi:10.1186/gb-2008-9-9-r137

11. Vikesaa J, Hansen TV, Jønson L, et al. RNA-binding IMPs promote cell adhesion and invadopodia formation. EMBO J. 2006;25(7):1456-1468. doi:10.1038/sj.emboj.7601039

12. Jiang W, Wang J, Zhang Y. Histone H3K27me3 demethylases KDM6A and KDM6B modulate definitive endoderm differentiation from human ESCs by regulating WNT signaling pathway. Cell Res. 2013;23(1):122-130. doi:10.1038/cr.2012.119

13. Naujok O, Diekmann U, Lenzen S. The generation of definitive endoderm from human embryonic stem cells is initially independent from activin A but requires canonical Wnt-signaling. Stem Cell Rev Rep. 2014;10(4):480-493. doi:10.1007/s12015-014-9509-0

14. Ninomiya H, Mizuno K, Terada R, et al. Improved efficiency of definitive endoderm induction from human induced pluripotent stem cells in feeder and serum- free culture system. In Vitro Cell Dev Biol Anim. 2015;51(1):1-8. doi:10.1007/s11626- 014-9801-y

15. Zhang J, Fei Y, Sun L, Zhang QC. Advances and opportunities in RNA structure experimental determination and computational modeling. Nat Methods. 2022;19(10):1193-1207. doi:10.1038/s41592-022-01623-y

16. Ma H, Jia X, Zhang K, Su Z. Cryo-EM advances in RNA structure determination. Signal Transduct Target Ther. 2022;7(1):58. Published 2022 Feb 23. doi:10.1038/s41392-022-00916-0

17. Conway AE, Van Nostrand EL, Pratt GA, et al. Enhanced CLIP Uncovers IMP Protein-RNA Targets in Human Pluripotent Stem Cells Important for Cell Adhesion and Survival. Cell Rep. 2016;15(3):666-679. doi:10.1016/j.celrep.2016.03.052

18. Zambelli F, Pavesi G. RIP-Seq data analysis to determine RNA-protein associations.

Methods Mol Biol. 2015;1269:293-303. doi:10.1007/978-1-4939-2291-8_18

**2nd round**

**Reviewer 1**

The lncRNA HIDEN was a newly characterized lncRNA during human PSC differentiation whose transcriptional position is more than 50 kb away from the protein coding region, classified as "desert lncRNAs". The authors utilized RNA pull-down assay to identify IGF2BP1 (IMP1) as the key RNA binding protein that interacting with HIDEN and controls the endoderm differentiation by stabilizing FZD5 mRNA to promote WNT activity.
The author has given reasonable explanations and replies to the questions of the two reviewers.

**Reviewer 2**

The authors have carefully addressed all comments. I have no further comments.
